# Multi-omics analysis to uncover the molecular basis of tumor budding in head and neck squamous cell carcinoma

**DOI:** 10.1038/s41698-025-00856-2

**Published:** 2025-03-13

**Authors:** Iordanis Ourailidis, Fabian Stögbauer, Yuxiang Zhou, Susanne Beck, Eva Romanovsky, Stephan Eckert, Barbara Wollenberg, Markus Wirth, Katja Steiger, Bernhard Kuster, Olivier Gires, Albrecht Stenzinger, Peter Schirmacher, Wilko Weichert, Peer-Hendrik Kuhn, Melanie Boxberg, Jan Budczies

**Affiliations:** 1 Institute of Pathology, University Hospital Heidelberg, Heidelberg, Germany; 2 Faculty of Biosciences, University of Heidelberg, Heidelberg, Germany; 3 Institute of Pathology, School of Medicine, Technical University of Munich, Munich, Germany; 4 German Cancer Consortium (DKTK), Partner Site Munich, a Partnership Between DKFZ and University Center Technical University of Munich, Munich, Germany; 5 German Cancer Research Center (DKFZ), Heidelberg, Germany; 6 Proteomics and Bioanalytics, School of Life Sciences, Technical University of Munich, Freising, Germany; 7 Department of Otolaryngology Head and Neck Surgery, School of Medicine, Technical University of Munich, Munich, Germany; 8 Comparative Experimental Pathology, School of Medicine, Technical University of Munich, Munich, Germany; 9 Bavarian Cancer Research Center (BZKF), Munich, Germany; 10 Clinic and Polyclinic for Otorhinolaryngology, Ludwig Maximilian University of Munich, Munich, Germany; 11 Center for Personalized Medicine (ZPM), Heidelberg, Germany; 12 Institute of Pathology Kaufbeuren Memmingen Ravensburg, Kaufbeuren, Germany

**Keywords:** Biomarkers, Head and neck cancer, Cancer genomics

## Abstract

Tumor budding (TB) is a prognostic biomarker in HPV-negative and HPV-positive head and neck squamous cell carcinoma (HNSCC). Analyzing TCGA and CPTAC mutation, RNA, and RPPA data and performing proteomics and IHC in two independent in-house cohorts, we uncovered molecular correlates of TB in an unprecedentedly comprehensive manner. *NSD1* mutations were associated with lower TB in HPV-negative HNSCC. Comparing budding and nonbudding tumors, 66 miRNAs, including the miRNA-200 family, were differentially expressed in HPV-negative HNSCC. 3,052 (HPV-negative HNSCC) and 360 (HPV-positive HNSCC) RNAs were differentially expressed. EMT, myogenesis, and other cancer hallmarks were enriched in the overexpressed RNAs. In HPV-negative HNSCC, 88 proteins were differentially expressed, significantly overlapping with the differentially expressed RNAs. CAV1 and MMP14 protein expression investigated by IHC increased gradually from nonbudding tumors to the bulk of budding tumors and tumor buds. The molecular insights gained support new approaches to therapy development and guidance for HNSCC.

## Introduction

Squamous cell carcinoma of the head and neck (HNSCC) is the sixth most common cancer worldwide and one of the leading causes of cancer-related deaths^1,2^. Tumor budding (TB), defined as the detachment of clusters of four or fewer tumor cells from the main tumor mass that infiltrate into the adjacent stroma^3,4^, is a morphological marker associated with aggressive tumor biology and unfavorable clinical outcome in several squamous cell carcinomas, including HNSCC and squamous cell carcinomas of the lung, but also in adenocarcinomas, e.g., of the colorectum^3,5–9^. TB is associated with nodal and distant metastases, early relapse, and poor survival^10–16^.

Epithelial-to-mesenchymal transition (EMT) describes the loss of epithelial characteristics of cells in favor of a more mesenchymal phenotype^17,18^. In tumors, EMT correlates with tumor cell dissemination and invasion^19^. Recent research shows that EMT in HNSCC is not a binary switching from an epithelial to a mesenchymal gene expression program, but is better described as a continuous transition including gene expression signatures exhibiting both epithelial and mesenchymal properties to various extents (as reviewed in ref. ^20^)—this spectrum is often referred to as partial EMT (p-EMT)^19^.

TB has been described as a morphological correlate of EMT in colorectal cancer and across cancer types^5,21^. The upregulation of several transcription factors (TFs), including the EMT-TFs SNAIL and SLUG, as well as of genes included in mesenchymal transcription programs (e.g., *VIM*, *PDPN*, and *MMP*s) were reported in parallel to the downregulation of genes involved in epithelial differentiation (e.g., cytokeratins, integrins)^3^. Among all canonical EMT-TFs, SLUG is the only TF associated with p-EMT signatures in several HNSCC cohorts, and SLUG-associated p-EMT prognosticates overall survival^22,23^. In oral squamous cell carcinomas, an increased protein expression of vimentin, TGF-β and β-catenin, and a decreased expression of E-cadherin was suspected to render tumor buds an EMT-phenotype^6^. However, the majority of studies on correlation of TB with EMT focused on single genes, were based on immunohistochemistry, and/or were performed on colorectal cancer^5,21^.

For locoregionally confined HNSCC, surgery, in some instances combined with radiotherapy (RT) and chemotherapy (CT), stays the therapeutic mainstay^1^. For locally advanced HNSCC, cetuximab combined with RT or the longer-in-use cisplatin-based radiochemotherapy are standards of care with demonstrated improved efficacy over RT alone^24,25^. In a recent phase II trial including patients unfit for cisplatin, immune checkpoint inhibitors (ICI) concomitant with RT did not improve tumor control compared to cetuximab-RT, but appeared less toxic^26^. As different treatment regimes are available for locally advanced HNSCC, the development of predictive biomarkers could contribute to better therapy guidance in this setting. For recurrent or metastatic HNSCC, ICI with or without CT has replaced the earlier standards of care following the Keynote-048 and CheckMate 141 studies^27,28^. Nevertheless, the prognosis remains poor for the advanced stages of HNSCC calling for the development of new therapies. The aim of the current study is to uncover the molecular underpinnings of TB, which is a hallmark of tumor aggressivity and progression, to support the development of predictive biomarkers and the identification of therapeutically exploitable targets.

To our knowledge, the molecular basis of TB in HNSCC has not yet been investigated in a comprehensive way that includes various molecular levels and large cohorts of budding and nonbudding tumors^5,29,30^. To fill this gap, we generated and analyzed TB and multi-omics data of HPV-negative and HPV-positive HNSCC. First, we analyzed the association of TB with mutations, miRNA data, whole transcriptome data, and reverse-phase protein array (RPPA) protein data of the TCGA HNSCC cohort (referred to as TCGA-HNSC). Second, we performed immunohistochemistry (IHC) of two selected proteins and liquid chromatography mass spectrometry-based (LC-MS-based) proteomics in two HNSCC cohorts from the Technical University of Munich (TUM). We screened our findings for genes and signatures with the potential to be developed further into prognostic or predictive markers for HNSCC.

## Results

**Fig. 1:**
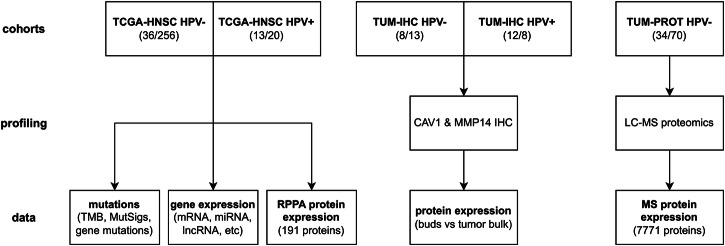
Overview of the study cohorts, -omics profiling platforms, and resulting data. We analyzed mutation, miRNA expression, mRNA expression and RPPA protein expression data from the TCGA HNSCC cohort (TCGA-HNSC). In addition, a first in-house HNSCC cohort (TUM-IHC) was analyzed for IHC for CAV1 and MMP14 and a second in-house HNSCC cohort (TUM-PROT) was analyzed by LC-MS-based proteomics. Only samples with histopathologically confirmed HNSCC and evaluable TB were included in the analyses. Sample sizes refer to the numbers of non-budding and budding tumors.

The study cohorts included the TCGA-HNSC dataset and two additional HNSCC cohorts profiled by IHC and LC-MS-based proteomics at the TUM (Fig. 1 and Supplementary Table 1). Furthermore, the CPTAC-HNSCC cohort was used as an additional validation cohort (Supplementary Table 1). In all these cohorts, tumor budding was evaluated by two board-certified pathologists analyzing the digitized H&E slides as detailed in Stögbauer et al.^8^ and in “Methods”. Primarily, we sought to compare molecular data between budding (any number of tumor buds detected) and non-budding (no tumor buds detected) tumors. Secondarily, we sought to analyze the correlation of molecular markers with the level of TB. We analyzed four cohorts of HPV-negative HNSCC (Supplementary Table 1): TCGA (292 tumors, 88% with TB), CPTAC (89 tumors, 74% with TB), the Munich cohort TUM-LC-MS (104 tumors, 70% with TB), and another Munich cohort of selected cases TUM-IHC (21 tumors, 62% with TB). We analyzed two cohorts of HPV-positive HNSCC (Supplementary Table 1): TCGA (33 tumors, 61% with TB) and a Munich cohort of selected cases TUM-IHC (20 tumors, 40% with TB). The prevalence of TB is dependent on the clinicopathological cohort characteristics, and the results for the investigated cohorts are in line with the values reported in the literature^6,8,11,31^.

### Analysis of mutations

Starting from the TCGA mutation calls, tumor mutational burden (TMB) was calculated as the total number of missense mutations. Levels of tumor mutational burden (TMB) did not significantly differ between budding and nonbudding tumors of the TCGA cohort, neither in HPV-negative nor in HPV-positive HNSCC. In addition, the single base substitution (SBS) mutational signatures were extracted using FitMS^32^. The mutational signatures SBS1, SBS2, SBS3, SBS4, SBS5, SBS13, SBS1, and SBS94 were detected in at least five HPV-negative HNSCC samples and the mutational signatures SBS1, SBS2, and SBS13 in at least 5 HPV-positive HNSCC samples. SBS18, connected to damage by reactive oxygen species, was significantly lower in budding compared to non-budding tumors in the HPV-negative subcohort (FC = −2.4, *P* = 0.005). The levels of all other analyzed mutational signatures did not significantly differ between budding and non-budding tumors, neither in HPV-negative nor in HPV-positive HNSCC.

**Fig. 2:**
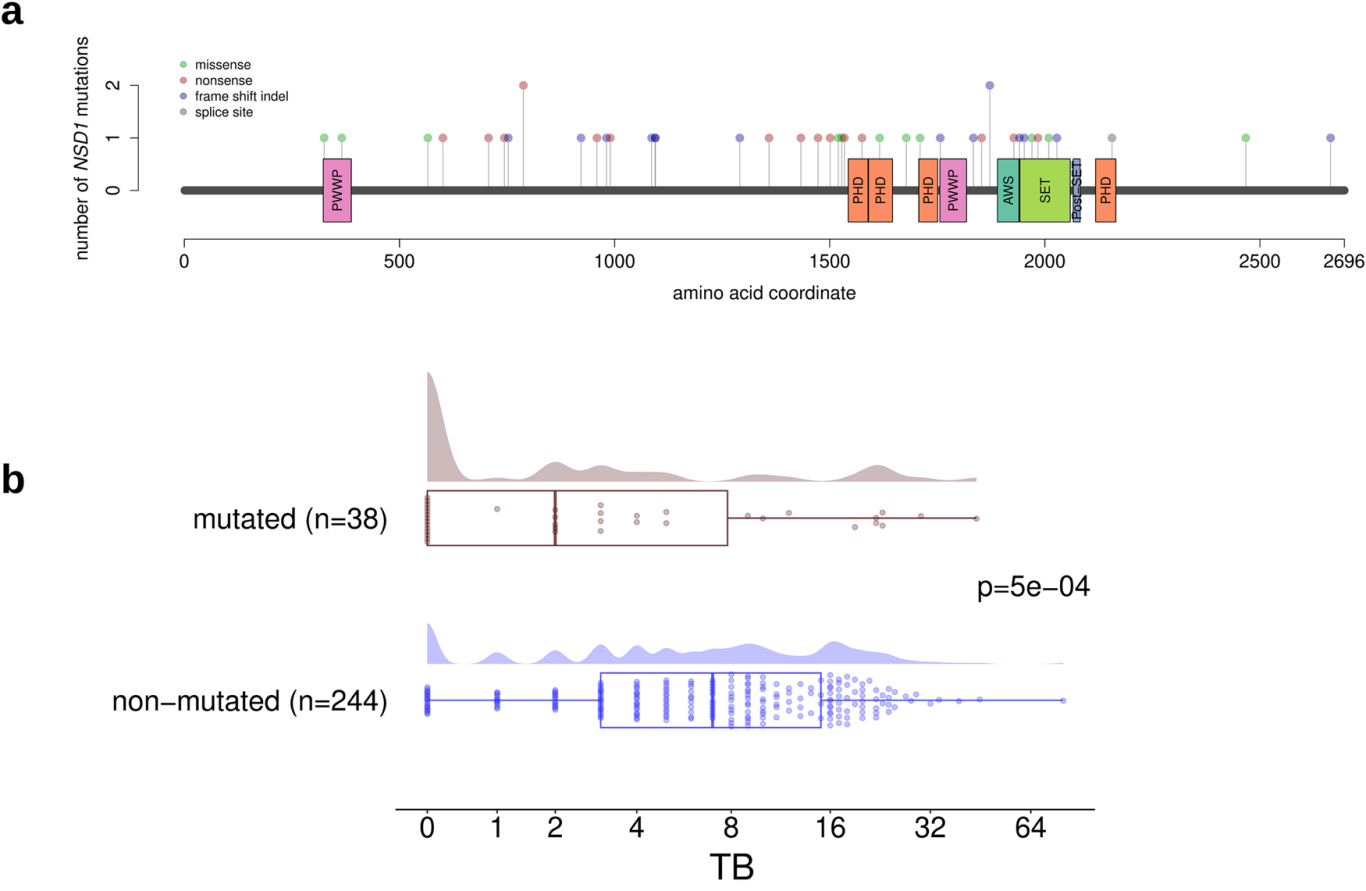
Analysis of *NSD1* mutations in HPV-negative HNSCC (TCGA-HNSC data). **a** Lollipop plot showing the location of the mutations in the *NSD1* gene. **b** Significantly higher TB in *NSD1*wt compared with *NSD1*-mut tumors.

Investigating the genes that were mutated in at least 10% of the tumors in the TCGA cohort, we detected a single significant association with TB; *NSD1* mutations were associated with lower TB in the HPV-negative subcohort (Fig. 2). A high proportion (72%) of the detected *NSD1* mutations were truncating mutations (frame-shift indels or nonsense mutations) and most probably associated with loss of function of the mutated protein. Analyzing TB as a dichotomized variable, the absence of TB was more frequent in *NSD1*-mutated compared to non-mutated *NSD1* tumors (36% vs. 9%, *P* = 2e-05). Analyzing TB as a continuous variable, TB levels were lower in *NSD1*-mutated compared to *NSD1* wild type tumors (FC = −1.41, *P* = 5e-04). Analyzing the clinicopathological tumor characteristics, *NSD1* mutations were more frequent in tumors of smokers (*P* = 0.002), associated with the absence of nodal metastasis (*P* = 0.002), and associated with tumor localization in the larynx (*P* = 0.0001, Supplementary Table 2). Bivariate logistic regression showed that *NSD1* mutation (*P* = 0.017) and TB (*P* = 0.0009) were independent predictors of nodal metastasis.

### Analysis of molecular subtypes

We analyzed the relation of TB with the previously validated gene expression subtypes of HNSCC^33,34^ and detected a significant association in the HPV-negative, but not in the HPV-positive subcohort. The atypical (at) HPV-negative cases exhibited the lowest (on average) tumor budding score, significantly lower than the mesenchymal (mes) and basal (b) HPV-negative cases (FC_b/at_ = 2.5, p_b/at_ = 0.004 and FC_mes/at_ = 3, p_mes/at_ = 0.002, Supplementary Fig. 1a). Similar trends were observed in the TCGA-HNSC HPV-positive cohort, although statistical significance was not reached, probably due to the low number of cases with non-atypical molecular subtypes (Supplementary Fig. 1b).

### miRNA analysis

TCGA-HNSC miRNA data were available for 289 (36 nonbudding) HPV-negative and 32 (12 nonbudding) HPV-positive cases and included 743 miRNAs. We identified 66 miRNAs differentially expressed between the budding and non-budding cases in HPV-negative HNSCC (Supplementary Fig. 2). By contrast, no differentially expressed miRNAs were identified in HPV-positive HNSCC, potentially due to the lower statistical power in this subgroup. Regulation of the miR-200 family consisting of 5 members (miR-200a, miR-200b, miR-200c, miR-141, miR-429) is known to be associated with tumor initiation, cancer progression, and EMT^35^. In HPV-negative HNSCC, all five members of the miR-200 family were significantly downregulated in the budding tumors with fold changes between −1.39 and −1.85. For miR-200 and miR-141, only the 5p arms were differentially expressed, while the 3p arms were not.

### Transcriptome analysis

**Fig. 3:**
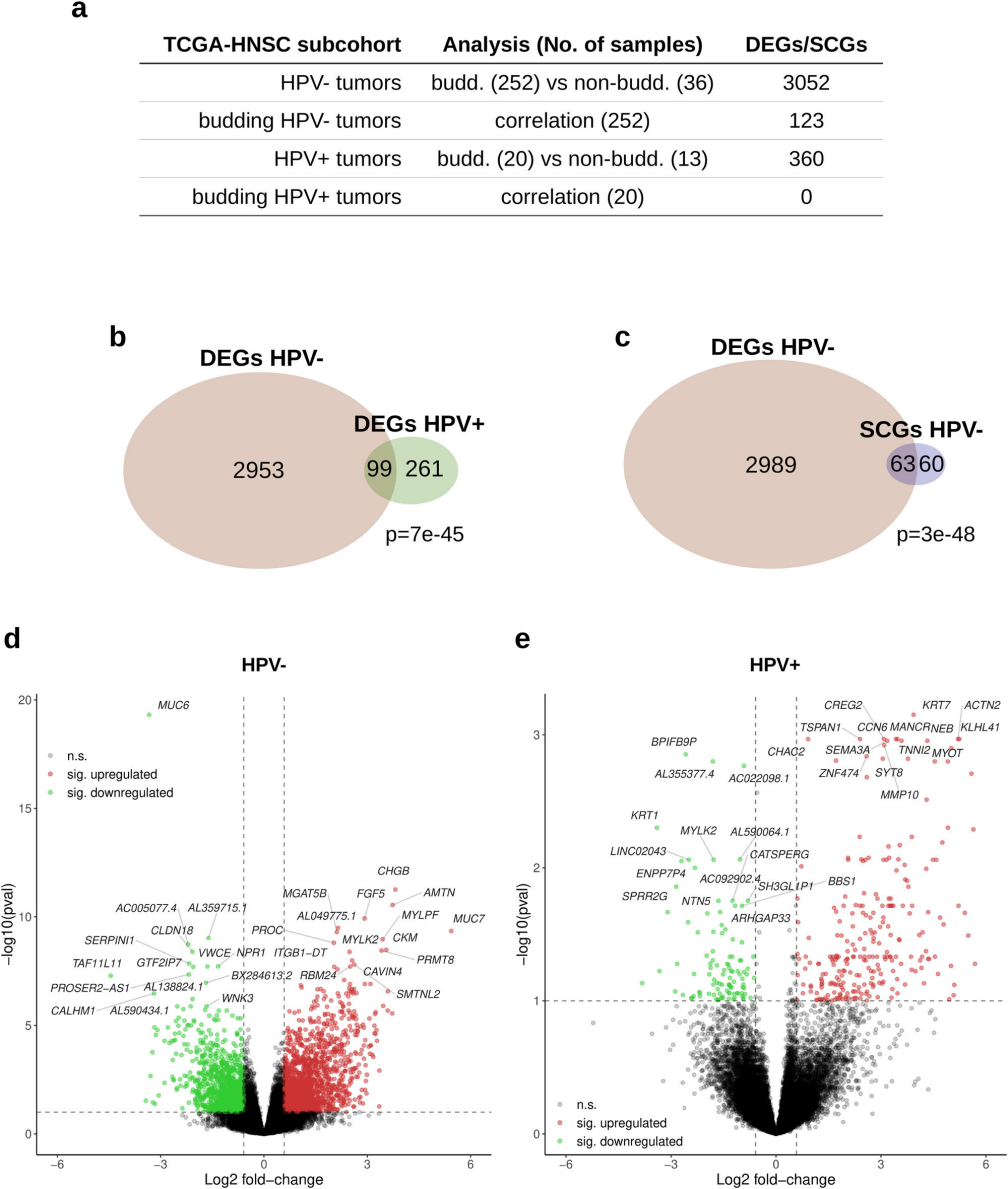
Analysis of differential mRNA expression between budding and nonbudding tumors (TCGA-HNSC data). **a** Overview of the performed analyses. **b** Intersection of the sets of DEGs detected in HPV- and in HPV + HNSCC. **c** Intersection between the DEGs and SCGs detected in the HPV- HNSCC. **d** Volcano plot showing the significantly upregulated (red) and downregulated (green) DEGs in the HPV-negative HNSCC (“pval” refers to the adjusted *P* values). **e** Same as (**d**), but for HPV-positive HNSCC.

For the detection of differential expressed genes (DEGs) and significantly correlating genes (SCGs) in the TCGA-HNSC gene expression data, we conducted two sets of analyses; a differential gene expression analysis between budding and non-budding tumors and a correlation analysis of each gene’s expression level with the extent of TB, excluding the non-budding cases. Each analysis was performed separately for the HPV-negative and the HPV-positive subcohort (Fig. 3). We identified 3052 DEGs (|FC | ≥1.5 at FDR 10%) in the HPV-negative subcohort (Fig. 3a, d) and 360 DEGs in the HPV-positive subcohort (Fig. 3a, e). In addition, we detected 123 SCGs (|*ρ* | ≥ 0.25 at FDR 10%) in the HPV-negative budding subcohort (Fig. 3a). Intersections of DEGs in the HPV-negative and HPV-positive TCGA-HNSC (*P* = 7e-45, Fig. 3b), as well as of the HPV-negative TCGA-HNSC DEGs and SCGs were significant (*P* = 3e-48, Fig. 3c). The top 15 upregulated and top 15 downregulated genes are shown in Fig. 3d (HPV-negative TCGA-HNSC) and Fig. 3e (HPV-positive TCGA-HNSC). To further validate our HPV-negative transcriptome-based findings, we used the CPTAC cohort and performed a differential gene expression analysis, using the same methodology. We identified 1504 DEGs between the budding and non-budding tumors (|FC | ≥1.5 at FDR 10%), and we could validate 858 (28%) of our HPV-negative TCGA-HNSC DEGs using the CPTAC-HNSCC cohort.

### Functional analysis

**Fig. 4:**
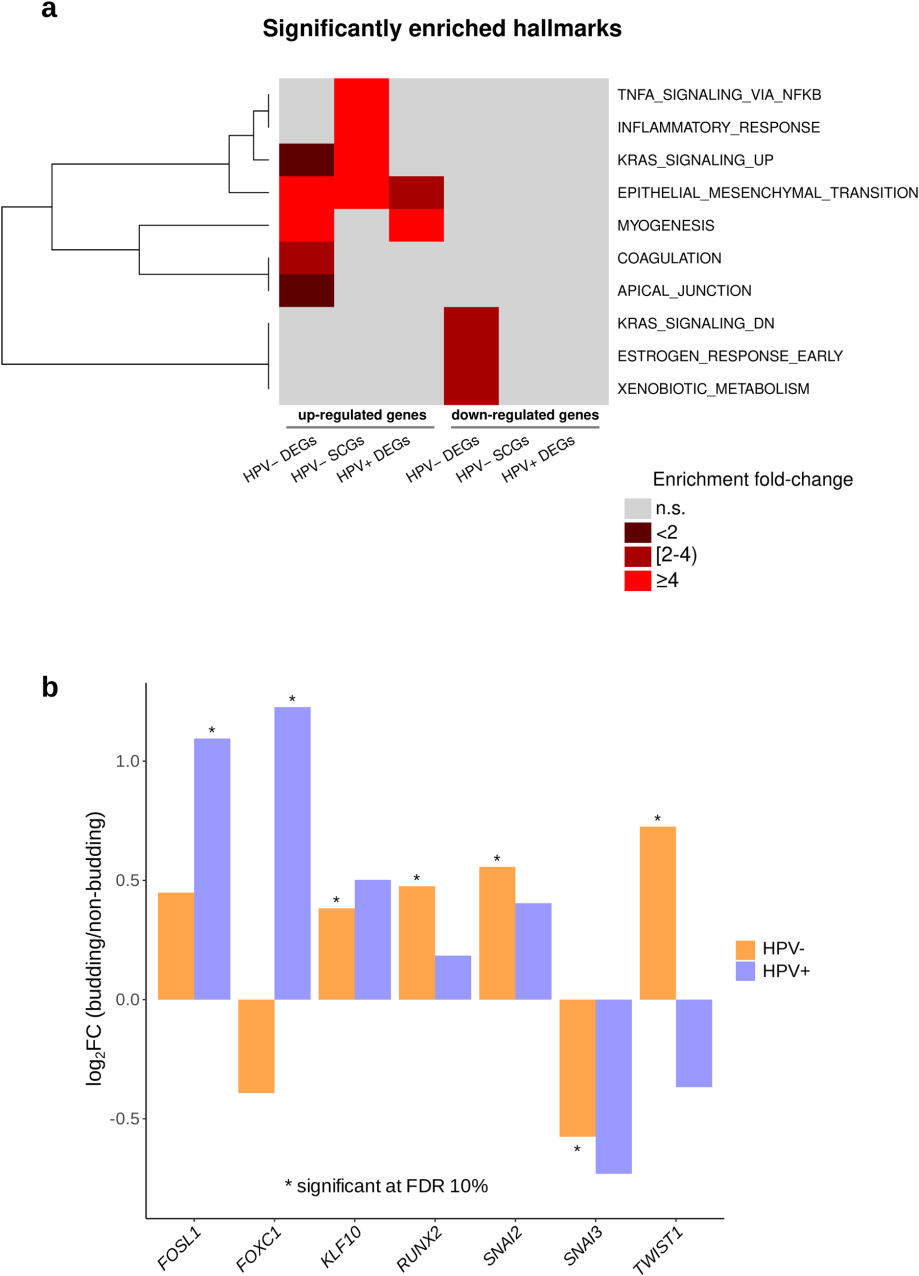
Functional analysis of the sets of DEGs and SCGs. **a** Enrichment analysis of the sets of DEGs and SCGs with respect to the 50 categories in the cancer hallmark catalog (MSigDB v.7.5). **b** Significantly up- and downregulated transcripts in a set of 36 analyzed EMT transcription factors.

To gain functional insight, the sets of TCGA-HNSC DEGs and SCGs were analyzed for enrichment of the hallmark gene sets in the MSigDB v7.5 database. Two enrichment analyses were performed for each of the subcohorts shown in Fig. 4a; one using the upregulated DEGs/positively correlating SCGs and one using the downregulated DEGs/negatively correlating SCGs. The upregulated DEGs/positively correlating SCGs of all subcohorts were significantly enriched in epithelial–mesenchymal transition (EMT) genes with a fourfold (*P* = 2e-22), eightfold (*P* = 1.7e-5), and threefold (*P* = 3e-4) enrichment fold change values for the HPV-negative DEGs, HPV-negative SCGs, and HPV-positive DEGs, respectively (Fig. 4a). Furthermore, the upregulated DEGs in the HPV-negative and HPV-positive subcohorts were significantly enriched in myogenesis genes (MSigDB hallmark gene set, Fig. 4a) with a fivefold (*P* = 4.8e-32) and an eightfold (*P* = 5.5e-18) enrichment fold change respectively. In addition, the downregulated HPV-negative DEGs were enriched for the “KRAS signaling (down)”, “estrogen response (early)”, and “xenobiotic metabolism” gene sets (Fig. 4a). Moreover, we could validate the enrichment of the EMT (fourfold, *P* = 1.7e-05), as well as the KRAS signaling (down) (fourfold, *P* = 4e-04) and coagulation (fourfold, *P* = 0.003) pathways using the upregulated DEGs of the CPTAC-HNSCC cohort.

To uncover the mechanism of EMT regulation, we performed an in-depth analysis of 36 EMT-associated transcription factors (Fig. 4b) using a previously published gene list^36^. *TWIST1*, *SNAI2*, *RUNX2*, and *KLF10* were overexpressed in the HPV-negative HNSCC tumors, while *SNAI3* was underexpressed. *FOSL1* and *FOXC1* were overexpressed in the HPV-positive HNSCC tumors (Fig. 4b).

**Fig. 5:**
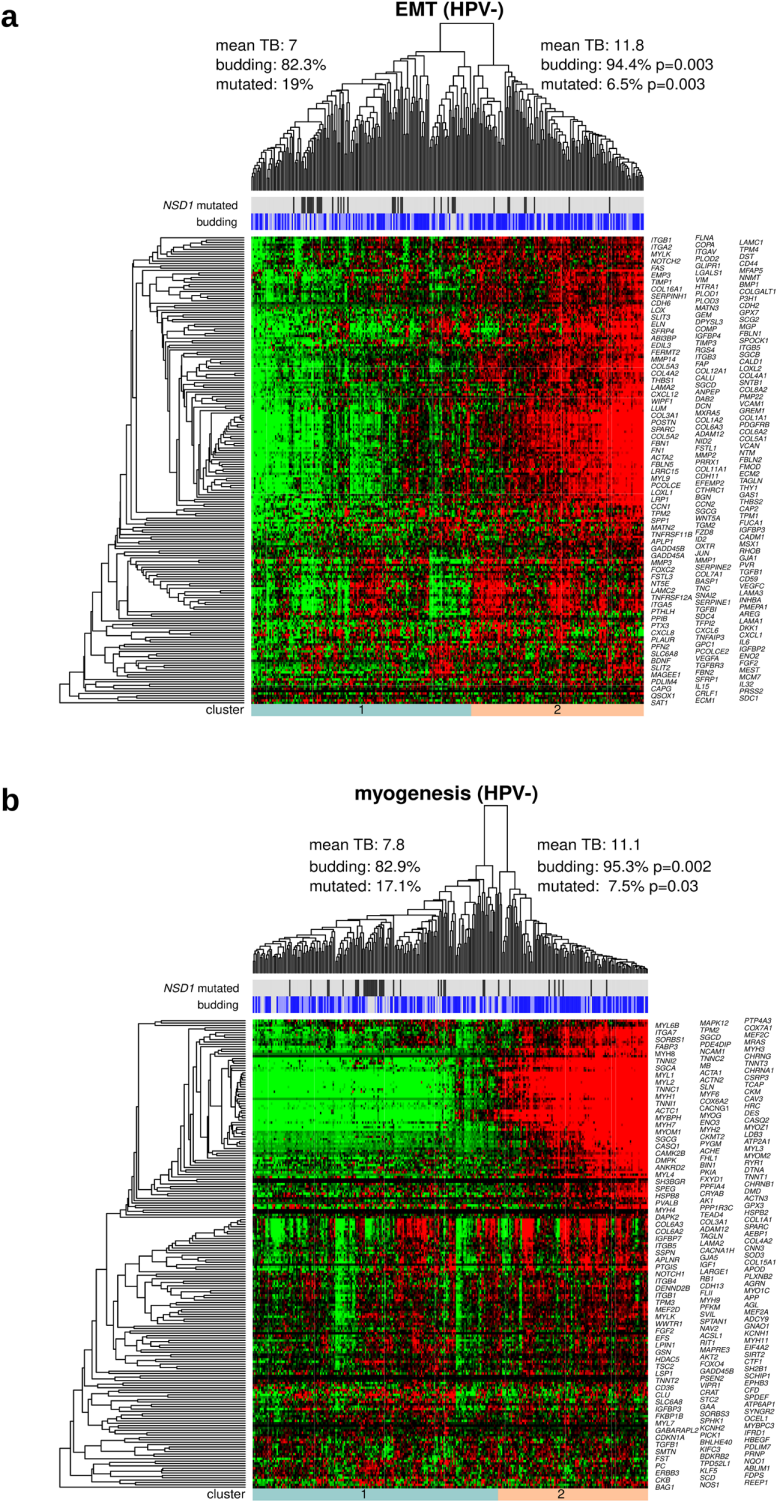
Unsupervised clustering analysis. **a** Clustering of HPV-negative tumors and genes with respect to the EMT category of the cancer hallmark catalog. **b** Same as (**a**), but for the myogenesis category. For both clusterings, TB and the proportion of *NSD1* mutations were significantly different between the two main tumor clusters.

To further investigate the gene expression patterns of the EMT and myogenesis hallmark gene sets, we performed an unsupervised hierarchical clustering analysis. Tumors were grouped according to the membership in the two clusters at the lowest level of the clustering hierarchy. In the HPV-negative subcohort (Fig. 5), the comparison of the two tumor clusters revealed a significant increase in the number of budding cases in the EMT-upregulated and myogenesis-upregulated clusters (*P* = 0.003 and *P* = 0.002, respectively) and as well as an increase in the number of *NSD1*-mutated cases in the EMT-downregulated and myogenesis-downregulated clusters (p = 0.003 and *P* = 0.03, respectively). Similar analyses in HPV-positive HNSCC did not lead to significant results, probably due to the small sample size of the HPV-positive subcohort (Supplementary Fig. 3). Heatmap analysis of the combined EMT and myogenesis gene sets resulted in three clusters in HPV-negative HNSCC; an EMT-downregulated and myogenesis-downregulated cluster, an EMT-upregulated and myogenesis-downregulated cluster, and an EMT-upregulated and myogenesis-upregulated cluster, with the latter exhibiting higher TB than the first two in the HPV-negative cohort (Supplementary Fig. 4). Interestingly, genes within the EMT and within the myogenesis gene sets showed similar gene expression patterns, while gene expression patterns were different between the two gene sets.

A landmark study investigating single-cell transcriptomics in HNSCC revealed a set of genes associated with a p-EMT gene expression program^22^. The top 15 “common” p-EMT genes (as defined in the publication) were selected for our downstream analysis. Remarkably, many of these genes in the gene expression program were included in the lists of TB-associated genes identified in the current study. In our TCGA-HNSC HPV-negative subcohort, all 15 genes of the gene expression program were included in the list of DEGs (*P* = 3.2e-20) and in our TCGA-HNSC HPV-positive subcohort, 5 genes of the gene expression program (*SERPINE1*, *MMP10*, *LAMC2*, *LAMA3*, and *INHBA*) were included in the list of DEGs (*P* = 2.0e-08).

We also performed hierarchical clustering with respect to a set of 22 epithelial, 15 mesenchymal markers, and HNSCC-specific p-EMT markers (Supplementary Figs. 5 and 6). In HPV-negative HNSCC, the cluster analysis resulted in five clearly separated tumor clusters, and the highest TB scores were observed in the clusters characterized by upregulation of mesenchymal markers regardless of the expression of epithelial markers (Supplementary Fig. 6).

### Immunohistochemical profiling

**Fig. 6:**
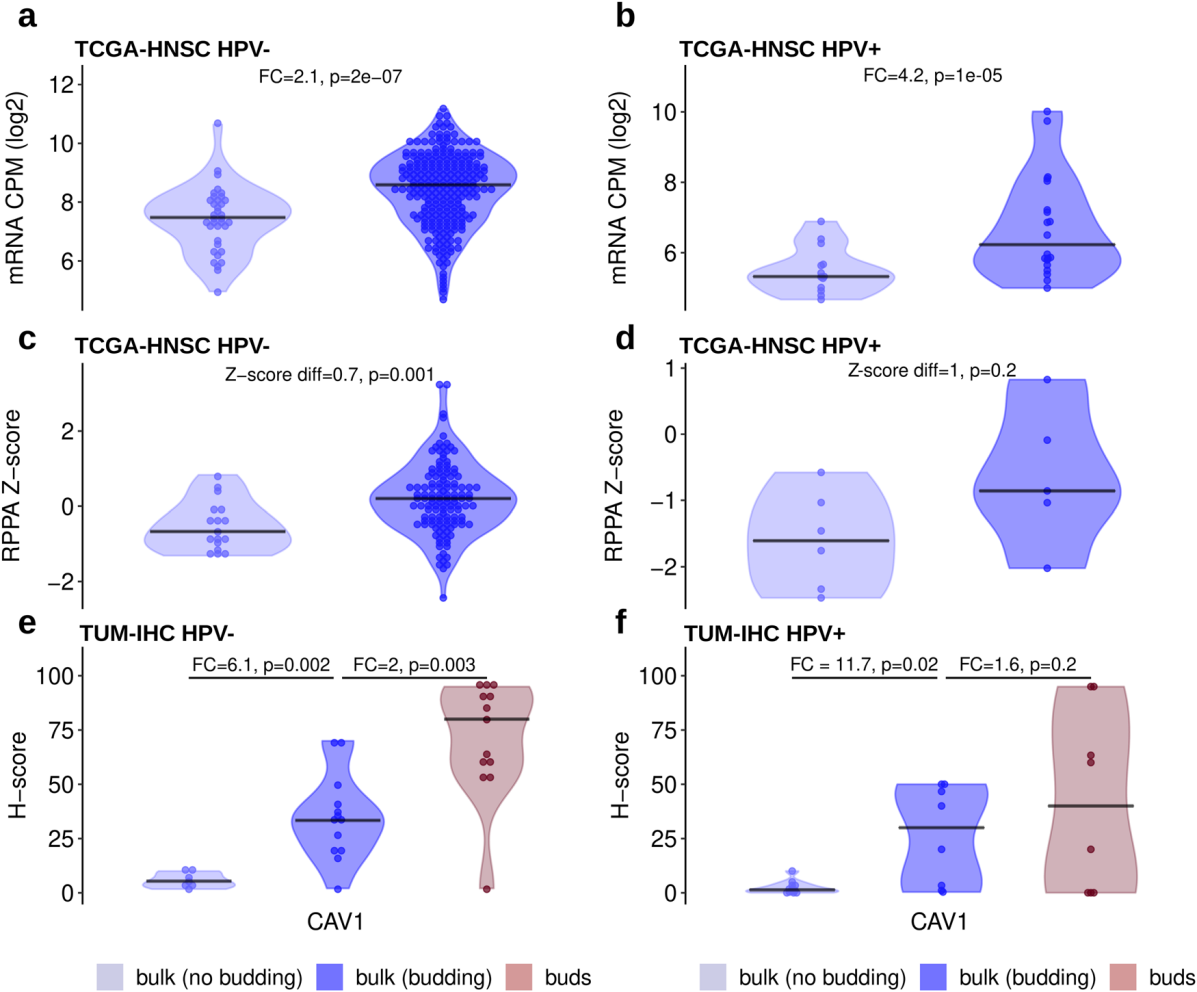
Inter- and intra-tumor analysis of CAV1 gene and protein expression in HPV-negative and HPV-positive HNSCC. **a** Increased mRNA expression in budding compared with non-budding HPV-negative tumors (TCGA-HNSC RNA-Seq data). **b** Same as (**a**), but for HPV-positive tumors. **c** Increased protein expression in budding compared with non-budding HPV-negative tumors (TCGA-HNSC RPPA data). **d** Same as (**c**), but for HPV-positive tumors. **e** Stepwise increasing protein expression from the bulk of non-budding tumors to the bulk of budding tumors to the buds of budding tumors in HPV-negative HNSCC (TUM-IHC cohort). **f** Same as (**e**), but for HPV-positive HNSCC.

The differential protein expression analysis of the RPPA TCGA-HNSC HPV-negative cohort revealed one protein, Caveolin-1 (CAV1), being upregulated in the budding cases (*P* = 0.001) (Fig. 6c). A similar trend can be observed in the HPV-positive cases (Fig. 6d) with an even greater difference between budding and non-budding cases, however, the difference is not statistically significant, potentially due to the small sample size. The upregulation of CAV1 in at least the HPV-negative cohort is in line with the upregulation of the *CAV1* mRNA in both the HPV-negative (*P* = 2e-07) and HPV-positive (*P* = 1e-05) subcohorts (Fig. 6a, b).

For validation of these results by an orthogonal experimental method as well as to gain insight in the cell type-specific and spatial distribution of protein expression, 20 HPV-negative and 20 HPV-positive tumors were stained for CAV1. A significantly higher H-score was observed in the tumor buds compared to the tumor bulk of the same cases in the HPV-negative subcohort (FC = 2, *P* = 0.003, Fig. 6e). At the same time, the tumor bulk of the budding cases showed significantly higher H-scores when compared to the tumor bulk of the non-budding cases in both the HPV-negative and HPV-positive subcohorts (Fig. 6e, f). Thus, there is an evident gradient of increasing CAV1 expression from the bulk of the non-budding cases, to the bulk of the budding cases, and finally to the tumor buds themselves.

In addition, we stained for MMP14, which was one of the highest upregulated genes in both the HPV-negative and HPV-positive TCGA-HNSC subcohorts, performed the same analysis as for CAV1, and observed similar results with a strong association of increased MMP14 expression in the tumor buds in both the HPV-negative and HPV-positive subcohorts (Supplementary Fig. 7).

### Proteome profiling

**Fig. 7:**
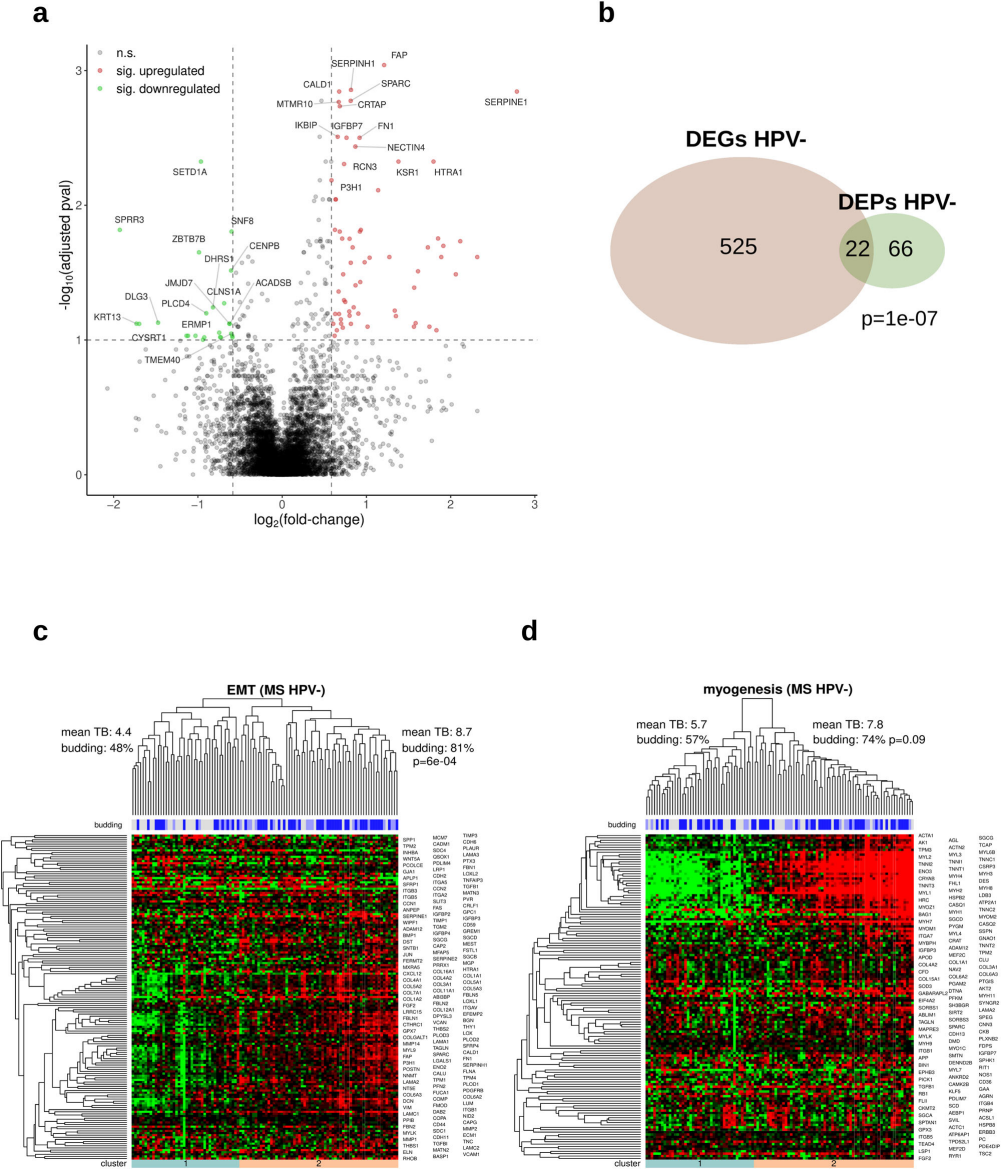
Analysis of differential protein expression between budding and non-budding HPV-negative tumors (TUM-LC-MS cohort). **a** Volcano plot showing the upregulated (red) and downregulated (green) DEPs (“pval” refers to the adjusted *P* values). **b** Intersection of the sets of detected DEPs and DEGs in HPV-negative HNSCC. **c** Unsupervised clustering of HPV-negative tumors and proteins with respect to the EMT category of the cancer hallmark catalog. **d** Same as (**c**), but for the myogenesis category.

LC-MS-based proteomics analysis was performed in a cohort of 104 HPV-negative HNSCC cases (70 budding and 34 non-budding). The proteome profiling resulted in the identification and quantification of 7771 proteins. Comparing the budding and non-budding cases, we detected 88 differentially expressed proteins (DEPs). Figure 7a shows the top 15 upregulated and top 15 downregulated proteins. There was a significant overlap between the DEGs detected during the TCGA-HNSC analysis (HPV-negative subcohort) and the DEPs of the in-house TUM-LC-MS cohort (*P* = 1e-07, Fig. 7b). The list of DEPs included two proteins (SERPINE1 and TNC) of the 15-gene-p-EMT gene list (*P* = 0.002), with 13 of the genes covered by the proteomics profiling. The enrichment analysis using the hallmark gene set of the MSigDB database and the DEPs confirmed our TCGA-HNSC findings, with the DEPs being enriched in EMT genes (enrichment FC = 9.9, *P* = 3e-15), coagulation genes (enrichment FC = 4.4, *P* = 0.002), and myogenesis genes (enrichment FC = 3.7, *P* = 0.005).

Hierarchical clustering with respect to the genes in the hallmark EMT gene set resulted in two clearly separated tumor clusters that strongly correlated with TB (81% vs. 48% of budding tumors, *P* = 6e-04, Fig. 7c). However, the same comparison was not significant for the hallmark myogenesis gene set (myogenesis-downregulated: 57% vs myogenesis-upregulated: 74%, *P* = 0.09, Fig. 7d). Furthermore, we utilized our in-house TUM-LC-MS DEPs and conducted a complementary protein expression analysis using the CPTAC-HNSCC protein expression cohort. This approach enabled us to validate 29 (36%) of the DEPs identified in the in-house TUM-LC-MS cohort.

### Network analysis

**Fig. 8:**
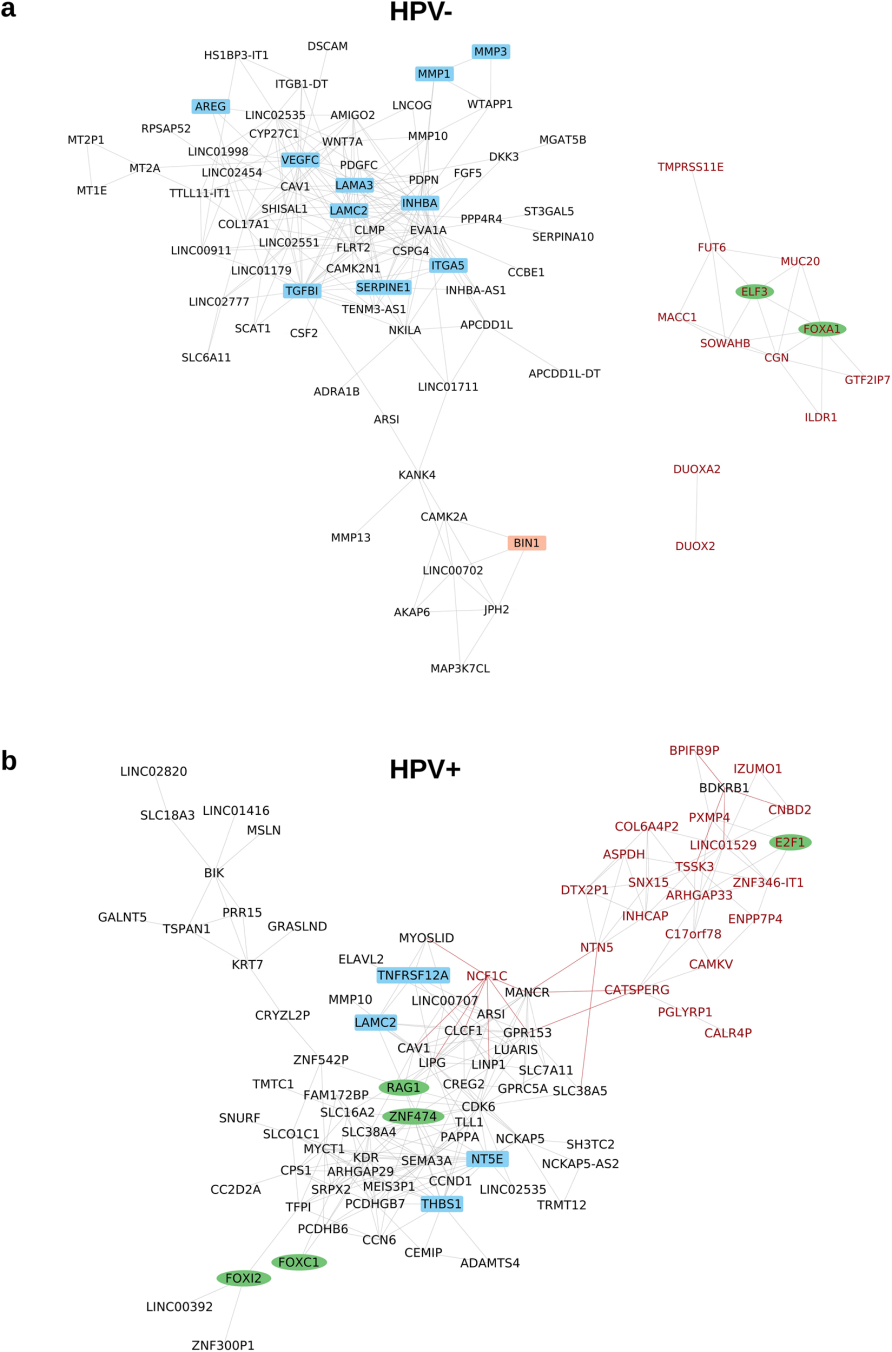
Correlation networks of the strong DEGs between budding and non-budding tumors (TCGA-HNSC data). **a** Network for HPV-negative HNSCC including 67 over- and 12 underexpressed genes. **b** Network for HPV-positive HNSCC including 68 over- and 22 underexpressed genes. Genes were included in the networks when being significant after multiple testing correction (FDR = 10%) and showing both |FC | ≥2 and AUC ≥ 0.7. Genes were connected by an edge when correlating with |Spearman *ρ* | ≥ 0.6. Black node font: overexpressed gene, red node font: underexpressed gene. Green ellipse: transcription factor, blue rectangle: EMT gene. Gray edge: positive correlation, red edge: negative correlation. Only genes correlating above the threshold with at least one other gene are shown.

To analyze the organization of the gene expression programs underlying TB in HNSCC, we generated correlation networks including the most strongly DEGs and DEPs (Fig. 8 and Supplementary Fig. 8). The network of DEGs included 67 overexpressed and 12 underexpressed genes for the TCGA-HNSC HPV-negative cohort, while it included 68 overexpressed and 22 underexpressed genes for the TCGA-HNSC HPV-positive cohort. Out of the overexpressed genes, *ARS1*, *CAV1*, *LAM2C*, *LINC02535*, and *MMP10* were shared by both networks, but none of the underexpressed genes. The oncogenic role of the long non-coding RNA LINC03535, its relation to EMT, and its negative association with OS have recently been elucidated in HNSCC and other cancer types^37^.

In HPV-negative HNSCC, the following 10 overexpressed genes were members of the hallmark EMT category: *AREG*, *INHBA*, *ITGA5*, *LAMA3*, *LAMC1*, *MMP1*, *MMP3*, *SERPIN1*, *TGFBI*, and *VEGFC*. *TGFBI* is induced by transforming growth factor-beta (TGFB) and encodes a protein inhibiting cell adhesion. In a recent preclinical study, an antibody targeting TGFBI with diagnostic and therapeutic potential was investigated in colorectal cancer models^38^. In these models, *TGFBI* silencing reduced tumor growth and angiogenesis. Based on these observations, blockage of *TGFBI* and other components of the TGFB signaling pathway warrants further investigation in HNSCC.

As blockade of EGFR signaling and ICI are the two main targeted treatment approaches in clinical use for HNSCC, we annotated the genes in the network by the gene ontology categories “immune response” (*n* = 1882) and “EGFR signaling pathway” (*n* = 123). A very low number of two (*CAV1* and *CSF2*) and five (*AV1*, *CLCF1*, *PGLYRP1*, *RAG1*, *THBS1*) immune genes were in the gene expression networks of HPV-negative and HPV-positive HNSCC, respectively. Moreover, none of the 14 immune cell populations identified from the RNA-Seq data by an earlier published method^39^ correlated with TB in any of the two subtypes. These two results support the view that TB and immune contexture are largely independent in HNSCC.

Of the genes contributing to EGFR signaling, amphiregulin (*AREG*) was the only one in the network of HPV-negative HNSCC, while *GPRC5A* was the only one in the network of HPV-positive HNSCC. The protein product of *AREG* is a ligand for the EGF/TGF-α receptor with connection to EMT, increased tumor cell migration and proliferation, and has been reported as a prognostic biomarker for treatment targeting EGFR in other cancer entities^40–43^. *AREG* should be further investigated as a potential biomarker that could be exploited for prognosis prediction and therapy guidance in HNSCC.

### Survival analysis

**Fig. 9:**
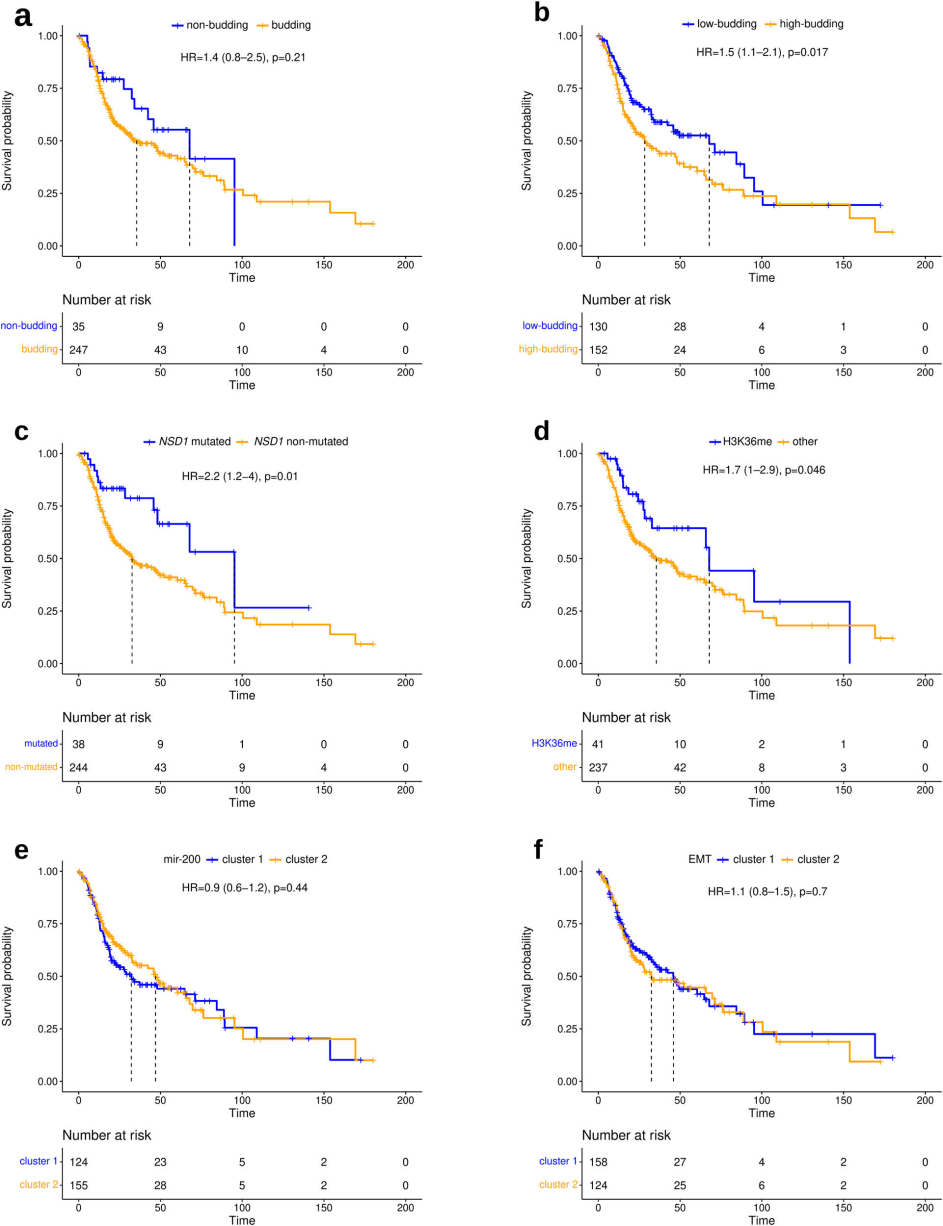
Analysis of OS (indicated in the month) in HPV-negative HNSCC (TCGA-HNSC cohort). **a** Comparison of budding and non-budding tumors. **b** Comparison of highly budding (TB ≥ 6) and lowly budding tumors (TB < 6). **c** Comparison of *NSD1*-mut and *NSD1*wt tumors. **d** Comparison of H3K36 methylated and non-methylated tumors. **e** Comparison of miR-200 family clusters 1 and 2. **f** Comparison of EMT clusters 1 and 2. For comparability, all markers are presented dichotomized to illustrate their respective prognostic impact on overall survival.

Performing multi-omics analyses, we identified various molecular correlates of TB, but observed imperfect correlations of the identified molecular markers and signatures with TB resulting in different ways to stratify HNSCC. To gain insight into the prognostic relevance of the different stratifications, analyses of overall survival (OS) and progression-free interval (PFI) were performed (Fig. 9 and Supplementary Figs. 9–11). In HPV-negative HNSCC, low TB, *NSD1* mutation, and impaired H3K36 methylation (a correlate of *NSD1* mutation^44^) were positive prognostic markers, while clustering of the tumors with respect to miRNA-200 and EMT-related mRNAs was not significantly associated with prognosis. In HPV-positive HNSCC, low TB was a positive prognostic marker, while impaired H3K36 methylation samples were not analyzed because they are uncommon in this subtype, and the miRNA-200 and EMT clusterings were again not significantly associated with prognosis. Among the two cutpoints investigated, TB reached statistical significance in HPV-negative HNSCC only for the higher cutpoint of 6, while TB reached statistical significance in HPV-positive HNSCC only for the lower cutpoint of 1.

The subset of HPV-negative HNSCC defined by *NSD1* mutation included 38 (13%) tumors, while the subset defined by H3K36 methylation impairment included 41 (15%) tumors. These subsets had a large and significant intersection of 28 tumors. The subset of non-budding tumors had a similar size (35 tumors, 12%), but the intersection with *NSD1*-mutated tumors was smaller (*n* = 14). The subset of low-budding tumors (cutpoint 6) was much larger and included 130 (46%) tumors, 28 of which were *NSD1*-mutated. For TB, all significant results were observed simultaneously for both investigated clinical endpoints. For *NSD1* and H3K36 in HPV-negative HNSCC, significance was reached only for OS, while it was narrowly missed for PFI.

## Discussion

We performed a comprehensive multi-omics analysis to uncover the molecular alterations underlying TB in HNSCC. We took the opportunity to analyze mutation, miRNA expression, and mRNA expression data available from the TCGA combined with TB data generated by the evaluation of the digital histopathological slides. Additionally, proteomics and IHC profiling were performed in in-house cohorts and proteomics data were available for the CPTAC cohort. All analyses were performed stratified for HPV status compliant with the distinction between HPV-associated and -independent HNSCC introduced in the WHO 2022 classification^45^. As previously published studies repeatedly generated evidence that tumor budding is associated with poor prognosis and predictive for lymph node metastasis in HPV-negative HNSCC^6,11,46^ and—as recently shown by our group—as well in HPV-positive oropharyngeal squamous cell carcinomas^8,31^, the main focus of the current study was to unravel the biology behind tumor budding on transcriptomic and proteomic level including spatially resolved analysis.

Analyzing tumor genetics, *NSD1* was the only gene that significantly correlated with TB in HPV-negative HNSCC, while no significant correlations were detected in HPV-positive HNSCC. In HPV-negative HNSCC, *NSD1*-damaging mutations were associated with lower TB and longer OS in line with earlier reports on a favorable prognosis of *NSD1*-mutated HPV-negative HNSCC^47,48^. *NSD1* is a member of the NSD family of histone methyltransferases which act as mono- and dimethyltransferases for H3K36^49^. Papillon-Cavanagh et al. identified a cluster of HNSCC with global DNA hypomethylation by unsupervised clustering of DNA methylation data^44^. The tumors in this cluster either had damaging alterations in *NSD1* or K36M-encoding mutations in one of the H3 genes and the authors concluded that it is defining a subtype of HNSCC with impaired H3K36 methylation. In the HPV-negative tumors of the TCGA, 68% of the tumors of this subtype had *NSD1* mutations. Here, we showed that HPV-negative tumors of the H3K36 subtype had numerically lower TB and significantly improved OS compared to other HPV-negative tumors. Altogether, these data support the view that tumors with impaired H3K36 methylation and in particular damaging *NSD1* mutations build a less aggressive tumor subtype of HPV-negative HNSCC less prone to metastasize. By contrast, the advantage of gaining *NSD1* mutations during tumor evolution is most probably due to crosstalk with immune-regulatory pathways resulting in immune exclusion^50,51^.

A high number (3052) of DEGs was detected between the budding and non-budding tumors of the TCGA-HNSC HPV-negative cohort. The protein products of 547 genes out of these were detectable by the in-house proteomics profiling in an independent cohort of HPV-negative HNSCC and 22 proteins could be confirmed to be differentially expressed. In addition, the proteomics analysis revealed 66 DEPs for which the corresponding mRNAs were not differentially expressed. The intersection of the finding of the transcriptome and proteome analyses was statistically significant and some of the differences might be explained by technical limitations including detection limits of the proteomics platform for the detection of lowly expressed proteins. Nevertheless, we believe that most findings of differential protein expression but unchanged mRNA expression reflect tumor biology, namely post-transcriptional regulation in the absence of transcriptional regulation. We also analyzed the correlation of mRNA expression with the level of TB. In this analysis, we excluded all non-budding tumors to strictly separate the presence and the extent of TB with possibly different underlying molecular processes. In the TCGA-HNSC HPV-negative cohort, we identified 123 SCGs, of which more than half were also differentially expressed, suggesting that similar biological processes drive both the initiation of TB and the regulation of its intensity.

Gene set enrichment analysis revealed EMT and myogenesis as the two most strongly enriched functional categories among the genes upregulated in budding HNSCC. A significant enrichment of these categories was observed in both the TCGA-HNSC HPV-negative and HPV-positive subcohorts. Correlation analyses of the DEGs annotated to the categories EMT and myogenesis revealed distinct gene expression patterns of the two categories in HNSCC (Supplementary Fig. 4). This observation supports the view that the two categories represent two independent biological processes that can be active or inactive in distinct sets of HNSCC rather than two annotations of a single biological process. The enrichment of genes annotated for EMT is in line with the broadly accepted view identifying tumor budding as the phenotype associated with EMT or p-EMT, the latter being a spectrum of cell states characterized by gaining mesenchymal marker while not completely losing the epithelial markers^19–21^. Myogenesis is the formation of muscle fibers, i.e., mesodermal tissue. Thus, genes annotated to EMT and myogenesis share the property to promote the production of mesenchymal cells. In line with our results, enhanced myogenesis in gastric cancer was recently reported to be associated with EMT, angiogenesis, and poor clinical outcomes^52^.

Downregulation of the miRNA-200 family has been implicated in cancer initiation, progression, and metastasis^53,54^. We detected downregulation of all five members of the miRNA-200 family in budding compared to non-budding HPV-negative HNSCC. This result is in line with earlier intra-tumoral analyses in oral and colorectal cancer showing downregulation of the miR-200 family in tumor buds compared to tumor bulk^55,56^. Mechanistically, members of the miR-200 family were found to directly repress the EMT-TFs *ZEB1* and *ZEB2* and uphold the epithelial phenotype, while their inhibition reduced *CHD1* expression, increased *VIM* expression, and induced EMT^57–59^. The results from cell culture experiments together with the observations in the current and earlier studies analyzing cancer tissues are in line with a contribution of reduced expression of the miRNA-200 family to EMT and in turn to TB in HPV-negative HNSCC by releasing the inhibition of *ZEB1/2* expression.

HPV status for the TCGA tumors was determined based on the RNA expression of the virus genes (Supplementary Fig. 12). By contrast, p16 IHC is typically used for this purpose in clinical routine diagnostics. For the TCGA cohort, p16 data are incomplete, while both HPV DNA and mRNA data are available. Published studies support the view that mRNA-based HPV classification monitoring active transcription of the virus genes most accurately reflects the unique clinical behavior and tumor biology of HPV-positive HNSCC^60,61^. While the three methods for HPV determination disagree for a significant proportion of tumors^61–63^, the RNA-based method, offering the cleanest separation with respect to tumor biology and clinical outcome, was used in the current study.

We observed a prognostic relevance of the TB level in HPV-negative and in HPV-positive HNSCC in line with earlier reports^8,64,65^. Of note, the definition of HPV status (based on detection of viral RNA) differed from the definition (based on the detection of viral DNA) used in an earlier analysis of the same TCGA dataset^8^. As described above, the RNA-based detection method focuses on the detection of tumors with active virus gene transcription and resulted in a smaller stratum of HPV-positive tumors and a larger stratum of HPV-negative tumors at the same time. Prognostic relevance was retained when using a TB cutpoint of 6 in HPV-negative HNSCC, but using a lower TB cutpoint of 1 in HPV-positive HNSCC. Of note, using the lower cutpoint, no progressions and no deaths were observed within the first four years after surgery in the HPV-positive stratum.

For a long time, it has been observed that the intensity of TB and the percentage of budding tumors are considerably lower in HPV-positive HNSCC compared with HPV-negative HNSCC. Some authors have argued that since HPV-positive HNSCC tumors typically do not exhibit tumor budding and TB-based stratification might be misleading in this subtype^13^. By contrast, two recent studies demonstrated and confirmed that the prognostic value of TB in HPV-negative HNSCC extends to HPV-positive HNSCC^8,31^. In line with these studies, we detected 360 DEGs between budding and non-budding HPV-positive tumors. Comparison with the results for HPV-negative HNSCC revealed that 99 genes were shared and that both lists of DEGs were enriched for the functional categories EMT and myogenesis. The molecular analysis supports the view that TB is a valid biomarker in HPV-positive HNSCC that should be further analyzed in the context of treatment guidance. Rather than patients with high-budding tumors, ones with non- or low-budding tumors should be preferred as candidates for treatment de-escalation.

Although correlating with TB, neither clustering of tumors with respect to the expression of the miRNA-200 family nor with respect to mRNA expression of EMT-related genes had prognostic relevance in HPV-negative and HPV-positive HNSCC. In HPV-negative HNSCC, a favorable prognosis was observed for the small (10–15%) subgroup of *NSD1*-mutated tumors. In HPV-positive HNSCC, no prognostic molecular markers that could replace TB were identified. As mutation analysis is not part of today’s routine diagnostic workflow for HNSCC tissue samples, the determination of TB from H&E tissue slides remains by far the easiest to implement and most cost-effective method among the investigated biomarkers for prognostication of HNSCC.

We performed bulk tissue transcriptomics and proteomics analyses to uncover inter-tumor differences associated with TB. While these analyses identified large sets of differentially expressed mRNAs and proteins, it is a limitation that these analyses do not address intra-tumor heterogeneity. Studies based on laser-capture microdissection to investigate tumor heterogeneity have uncovered intra-tumor expression changes between tumor buds and tumor bulk in HNSCC and other cancer types^55,56,66^. In concordance with the current results, the detected gene sets in the microdissection studies were enriched for EMT and other processes involved in invasion and metastasis. By contrast, the interpretation of the enrichment of lists of DEGs for myogenesis is less clear, as the differential gene expression could either arise from the tumor cells, the tumor microenvironment (TME), or both. A full understanding of inter- and intra-tumor heterogeneity of TB can only be gained by a spatially resolved analysis of both budding and non-budding tumors. In the current study, we performed such an analysis for CAV1 and MMP14 and observed a gradually increasing protein expression from the bulk of non-budding tumors to the bulk of budding tumors to the buds of budding tumors. These results support the view that both inter- and intra-tumor molecular differences contribute to TB. In line with our observations, *CAV1* has been reported to promote tumor cell migration and tumor invasion in various cancer types, including HNSCC, in cell lines, mouse models, and human patients^67–69^ and its association with cancer invasion and poor prognosis was connected to increased expression of matrix metalloproteinases in hepatocellular cancer^70^. These reports combined with our data suggest that high expression of *CAV1* is a marker of high TB, aggressive tumor behavior, and unfavorable prognosis in HNSCC. *NSD1* mutations are an example of a spatial constant tumor-specific determinant of TB that is not influenced by tumor heterogeneity. Spatial -omics technologies offer the opportunity to obtain a comprehensive spatially resolved portrait of TB including separate profiling of tumor cells and cells in the TME.

ICI and blockade of the EGFR signaling are the main therapeutic approaches currently in use for the treatment of HNSCC beyond surgery, RT, and CT. Tumor infiltrating lymphocytes and in particular T cells are now used as a prognostic marker for HNSCC patients not receiving ICI^71,72^, while B-cell infiltration was recently shown to be associated with the survival of ICI-treated HNSCC patients^73^. By contrast, TB did not correlate with the levels of immune cell populations in the current study. While CAV1 has been shown to regulate the organization of isotype-specific B-cell antigen receptors and in turn B-cell tolerance^73,74^, IHC analysis in the study cohort showed that CAV1 is implicated in TB via the expression on tumor cells, not on immune cells. These results support the view that TB and the contexture of the TME are largely independent factors in HNSCC. Analysis of the strongly overexpressed genes in budding tumors revealed amphiregulin (*AREG*) as the only gene implicated in EGFR signaling in HPV-negative HNSCC and *GPRC5A* as the only such gene in HPV-positive HNSCC. Preclinical models of *EGFR* wild-type HNSCC and lung cancer producing high levels of amphiregulin evaluated by ELISA were more likely to be growth inhibited in response to both gefitinib and cetuximab^75^. By contrast, patients with high amphiregulin evaluated by IHC had shortened OS and PFS in a phase II clinical trial of HNSCC patients receiving cetuximab and docetaxel^75,76^. Further investigation is needed to determine the optimal molecular level and measurement method for amphiregulin as a predictive biomarker, as well as strategies to inhibit the related autocrine activation of EGFR signaling in HNSCC.

In conclusion, our comprehensive multi-omics analysis of TB in HNSCC revealed associations on various molecular levels in both the HPV-negative and the HPV-positive subtype. While *NSD1* mutation in HPV-negative tumors was the only tumor genetic correlate, massively different miRNA, mRNA, and protein expression patterns were found to be related to the TB state, indicating that the molecular underpinnings of TB are more related to transcriptomic and proteomic signatures than to specific gene mutations. Network analysis revealed *AREG* and *TGFBI* as potential theragnostic markers in HNSCC that warrant further investigation. Altogether, the multifaceted insights gained contribute to a better understanding of the molecular processes driving TB in HNSCC and identified new potential opportunities for future therapeutic interventions in this devastating disease.

## Methods

### TCGA and CPTAC data

The TCGA-HNSC cohort consisted of 528 patients with a total of 471 digitized H&E-stained slides available from the GDC Data Portal (https://portal.gdc.cancer.gov)^77^. An additional 140 cases had to be excluded due to a different tumor entity, too small tissue fragments not allowing for the analysis of 10 high-power fields, an insufficient scan quality, duplicates, a history of neoadjuvant treatment, and cancer tissue without surrounding stroma precluding the analysis of tumor budding as described in Stögbauer et al.^8^. Similarly, the tumor budding status and intensity could be determined in 90 patients of the CPTAC-HNSCC cohort^78^.

Primary tumor TCGA-HNSC RNA-Seq BAM files were downloaded using gdc-tool v.1.6.1^77^. We calculated the number of reads mapped to the Human Papillomavirus (HPV) genome, as described in the TCGA-HNSC publication^61^, and normalized the read counts to counts per million (CPM). Two distinct clusters of samples were formed; samples with a HPV CPM sum of greater than 2 were considered HPV-positive and constituted the HPV-positive subcohort (*n* = 33), while the rest constituted the HPV-negative subcohort (*n* = 292) (Supplementary Fig. 12a). At the same time, we applied TCGA’s methodology of HPV status determination^61^, where cases with more than 1000 reads mapped to the Human Papillomavirus (HPV) genome were considered HPV-positive and the rest HPV-negative (Supplementary Fig. 12b); the classification of the samples (HPV-positive or HPV-negative) using normalized counts was identical to the classification using the TCGA-HNSC’s raw read count method. The HPV status of the CPTAC-HNSCC samples was extracted from Huang et al. 2021, where it was estimated in the same way as described above for the TCGA-HNSC cohort^79^. There was only one HPV-positive sample, which was omitted from the analyses, and the final CPTAC-HNSCC cohort consisted of 89 HPV-negative samples.

The TCGA-HNSC mutation data were downloaded from the TCGA Pan-Cancer analysis project^80^ (file: mc3.v0.2.8.PUBLIC.maf.gz, accessed on: 13.02.2024). Mutation data were available for 282 HPV-negative and 30 HPV-positive cases. Only non-synonymous mutations and mutations at splice sites were further considered.

TCGA-HNSC miRNA gene expression data (normalized) were downloaded from the Cancer Genome Atlas Pan-Cancer analysis project^80^ (file: pancanMiRs_EBadjOnProtocolPlatformWithoutRepsWithUnCorrectMiRs_08_04_16.csv, accessed on: 13.02.2024). Data were available for 289 (36 nonbudding) HPV-negative and 32 (12 nonbudding) HPV-positive cases and included 743 miRNA genes.

Primary tumor TCGA-HNSC and CPTAC-HNSCC RNA-Seq raw count data and transcript per million (TPM) values were downloaded using gdc-tool v.1.6.1^77^. The raw gene counts were additionally normalized to counts per million (CPM). Raw read count data and DESeq2^81^ were used to perform the differential gene expression analyses. The molecular subtypes atypical, classical, mesenchymal, and basal, as described in the HNSCC study by the TCGA^61^, were downloaded for available cases from the supplementary material of the TCGA-HNSC publication (Supplementary Data 7.2) and we investigated the tumor budding score differences in the aforementioned molecular subtypes. H3K36M HNSCC subgroup information was retrieved from Papillon-Cavanagh et al.^44^. Abundances of 14 immune cell populations from the TCGA RNA-Seq data were calculated using an earlier published method^39^.

TCGA-HNSC reverse-phase protein array (RPPA) normalized data were downloaded from cBioPortal (cohort: HNSC with 143 HPV-negative and 11 HPV-positive HNSCC samples profiled for 191 proteins; file https://cbioportal-datahub.s3.amazonaws.com/hnsc_tcga_pan_can_atlas_2018.tar.gz)^82^ and CPTAC-HNSCC mass spectrometry-based normalized protein expression values were downloaded from the LinkedOmics database (cohort: HNSC with 108 samples HPV-negative and 1 HPV-positive samples profiled for 11561 proteins; file: https://cptac-pancancer-data.s3.us-west-2.amazonaws.com/data_freeze_v1.2_reorganized/HNSCC/HNSCC_proteomics_gene_abundance_log2_reference_intensity_normalized_Tumor.txt)^83^. Data were available for 143 HPV-negative and 11 HPV-positive cases and included 191 proteins. Using the above data, we assessed the differences between the budding and non-budding cases in terms of miRNA gene expression and protein expression.

### Immunohistochemical profiling

The TUM-IHC cohort consisted of 41 resection specimens of HNSCC from patients treated at the Klinikum rechts der Isar of the Technical University of Munich (TUM). Analysis of the cohort was conducted in accordance with the Declaration of Helsinki and authorized by the ethics commision of the University Hospital Rechts der Isar (vote 2023-543-S-SB). The cohort was selected to include a similar number of HPV-negative and HPV-positive tumors and a similar number of budding and non-budding tumors for both subtypes. 13 patients (31.7%) were female, and the median age was 62 years (interquartile range 13.5 years). 21 (51.2%) of the tumors were located in the oral cavity and 20 (48.8%) located in the oropharynx. Applying the guidelines of the College of American Pathologists^84^, 20 (48.8%) tumors were immunohistochemically p16-positive, while the remaining 21 tumors were p16-negative. The TUM-IHC cohort of 21 HPV-negative (13 with tumor budding) and 20 HPV-positive (8 with tumor budding) HNSCC samples was stained for CAV1 (anti-CAV1 antibody #3238, 1:200, Cell signaling, detected with Bond Polymer Refine Red Detection, Leica) and MMP14 (anti-MMP14 antibody ab51074, 1:500, Abcam, detected with Bond Polymer Refine Detection, Leica) on Leica Bond RX. Supplementary Figure 13 presents examples of CAV1 and MMP14 IHC stains alongside corresponding H&E stains for non-budding cases, as well as bulk and budding regions of budding cases. One non-budding CAV1-stained sample was removed from the analysis (low quality).

A “histo-score” (H-score) was calculated for each sample. H-score is defined as the intensity of staining multiplied by the percentage (P) of cells staining negative (0), weak (1 +), moderate (2 +), and strong (3 +), giving an analytical range for 0 to 300^85^. For the budding cases, we calculated two H-scores; one for the tumor cells of the non-budding regions and one for the tumor buds. The resulting H-scores were divided by 3, leading to values ranging from 0 to 100.

### Proteomics profiling

The TUM-Proteomics cohort consisted of 104 HPV-negative HNSCC cases and is a subcohort of 157 treatment-naive cases of resection specimens of OSCC (tongue, floor of mouth) published earlier^64^. From the published cohort, we excluded 10 p16-positive cases as well as 43 cases for which tumor purity was too low or protein extraction was insufficient for downstream analysis. Analysis of the cohort was conducted in accordance with the Declaration of Helsinki and authorized by the ethics commission of the University Hospital Rechts der Isar (vote 296/17 S). TUM-Proteomics included 70 budding and 34 non-budding tumors that were profiled for 7771 proteins.

#### Tissue microdissection and protein extraction

Tumor microdissection was performed after marking of the tumor on an H&E-stained slide by experienced head and neck pathologists (MB). The percentage of vital tumor cells was documented and was >60% for all included samples. Subsequently, samples of FFPE tissue sections were cut in 5 µm and deparaffinized according to our optimized protocol. In detail, FFPE tissue sections were incubated in a heating oven for 30 min at 60 °C and subsequently deparaffinized in washing solutions as the order of 100% xylene for 20 min, 100% Ethanol, 96% Ethanol, 70% Ethanol and protease-free ddH_2_O for 10 min respectively. Tumor cells from the marked area were scratched from deparaffinized tissue and transferred to 1.5 ml polypropylene microcentrifuge tubes containing 400 µl protein extraction buffer consisting of 0.5 M Tris-HCl pH 9.0, 10 mM DTT, 4% SDS(w/v). After shortly spinning on a table vortex mixer, all samples were incubated in a Thermomixer (Eppendorf) for 1 h at 100 °C followed by shearing DNA and debris via sonication using a Bioruptor Pico (15 cycles, 1 min on and 30 s off). After sonication, the protein products were pelleted at 14,000 rpm for 10 min (Eppendorf, Centrifuge 5430 R). The supernatants were transferred in fresh 1.5 ml tubes and stored at −20 °C until the next step.

#### Protein purification and digestion for mass spectrometry analysis

Prior to tryptic digestion, the detergent was removed from lysates by SP3 cleanup, following the protocol as described^86^. Briefly, the lysate was mixed with SP3 beads and proteins were precipitated in a 50:50 mixture of Sera-Mag Speed Bead types A and B (Thermo Fisher Scientific) in 70% acetonitrile. Beads were washed twice with 80% ethanol in water and once with acetonitrile. Disulfide bonds were reduced with 10 mM TCEP (tris(2-carboxyethyl)phosphine) for 45 min at 37 °C, followed by alkylation of cysteines with 55 mM CAA for 30 min at room temperature in 50 μL of digestion buffer (2 mM CaCl_2_ in 40 mM Tris-HCl, pH 7.8). Trypsin [1:50 (wt/wt) enzyme-to-protein ratio] was added, and bead-precipitated proteins were digested at 37 °C for 3−4 h. The same amount of trypsin was added again, and the digestion was allowed to continue overnight. The next day, beads were settled using a magnet, and the supernatant was transferred to a new tube. Beads were washed by the addition of 50 μL water, sonicated (3 × 30 s), and the supernatants were combined. Samples were acidified with FA to pH <3. Subsequently, peptides were loaded onto Evotips. For the optimization of the extraction procedure, samples were desalted using a C18 SepPak 96-well desalting plate before measurement.

#### Whole proteome analysis MS method

Samples were analyzed as described previously^87^. Briefly, 2 × 600 ng of each sample were loaded onto two single-use trap columns (Evotips) according to the manufacturer’s protocol and analyzed on an Evosep One LC-system coupled to an Orbitrap Exploris 480 mass spectrometer (Thermo Fisher Scientific) equipped with a high-field asymmetric-waveform ion-mobility spectrometry (FAIMS) unit (Thermo Fisher Scientific). The peptides were separated on an Evosep C18 column (3-µm particle size, 150 µm inner diameter) using the 15 samples per day (SPD; 88 min) method. Each sample was analyzed twice with two different sets of compensation voltages (CVs; set1: −30 | − 40 | − 50 | − 60 | − 70 V, set2: −35 | − 45 | − 55 | − 65 | − 75 V). The mass spectrometer was operated in positive ionization (spray voltage of 2300 V) and data-dependent acquisition mode, automatically switching between MS1 and MS2 scans with a fixed cycle time of 3 s. MS1 spectra were acquired over a mass-to-charge (*m/z*) range of 360–1300 *m/z* at an Orbitrap resolution of 60,000 (at *m/z* 200) using a maximum injection time (maxIT) of 45 ms and a normalized AGC target value of 100% (1 × 10^6^). The monoisotopic precursor selection filter was activated and set to the peptide mode. The charge state filter was set to 2–6. For MS2, precursors were isolated with a width of 1.3 Th, collected using a standard maxIT of 25 ms and a normalized AGC value of 100% (1 × 10^5^), fragmented by HCD at 28% NCE, and the spectra were acquired on the Orbitrap at a standard resolution of 15,000 (at *m/z* 200). The dynamic exclusion duration of fragmented precursor ions was set to 90 s. The dynamic exclusion list was set to be shared across different experiments.

#### Proteomic data analysis

Analysis of raw data files including protein identification and label-free quantification was performed with Fragpipe (v18.0) using a reviewed human proteome Uniprot reference database (access on 24.11.2020) and the following search settings in Fragpipe: MBR ion FDR 0.5%, Min probability 0.5, Fragment mass tolerance 20 ppm, MBR peptide FDR 1%, MBR top runs 410. False discovery rate for peptide/protein identification was set to 1% on the peptide and protein level. Missing values for a given protein were defined as missing not at random (MNAR) when it was detected in more than 90% of or in less than 10% of all samples. Missing values of all other protein groups were defined as missing at random (MAR) and were imputed with the package missRanger (v2.2.1). The MNAR values were subsequently imputed with a row-wise imputation of a random distribution -5 log values away from the row minimum and with a range of plus the row standard deviation and minus the row standard deviation.

We investigated the difference between the budding and non-budding cases in terms of protein expression. In addition, within the budding group, we investigated the correlation between the tumor budding score and each protein’s expression. Functional analysis was performed as described below for the differentially expressed genes.

### Determination of tumor budding

For all three datasets, tumor buds were defined as clusters of up to four tumor cells dissociating from the tumor mass and infiltrating into the surrounding stroma^5,9^. TB was assessed as described in detail in Stögbauer et al.^8^. In short, TB was counted irrespective of the location within the tumor, i.e., tumor center or periphery and was documented as the absolute number of tumor buds in ten consecutive high-power fields (HPFs) with one HPF covering an area of 97,464 μm^2^ in digitized HE-stained slides, corresponding to a field diameter of 0.35 mm in light microscopy, starting with the HPF including the highest amount of buds (“hotspot”). TB was evaluated by two experienced pathologists (FS and MB). In case of ambiguous results, cases were discussed, and a consensus rating was reached. We did not analyze interobserver agreement for this study as previous studies already showed high interrater agreement for tumor budding scoring in squamous cell carcinomas^88,89^ and our focus for the current study was directed towards the molecular background of tumor budding. Diverging from the recommendations of the International Tumor Budding consensus conference for colorectal cancer, we evaluated a 40×-field and not a 20×-field, as we aimed to apply a methodology comparable to other studies carried out in squamous cell carcinomas^3,6,8^.

### Statistical analysis

TB was primarily analyzed as a dichotomized variable (budding vs. non-budding) and secondarily as a continuous variable.

Starting from the TCGA mutation calls, tumor mutational burden (TMB) was calculated as the total number of missense mutations as described previously^90^. Single base substitution (SBS) mutational signatures were extracted using FitMS with the R package signature.tools.lib v2.4.4^32^. Only SBS signatures detected in at least five samples were subjected to further analysis. The levels of TMB and of the SBS signatures were compared between budding and non-budding tumors using the Wilcoxon test. The association of all genes mutated in at least 10% of samples with TB was assessed using Fisher’s test and Wilcoxon test. Differences of TB across the molecular subtypes of HNSCC were assessed using the Kruskal–Wallis test and *post-hoc* pairwise comparisons with the Wilcoxon test. Differences of immune cell population abundances between the budding and non-budding cases were assessed with the Wilcoxon test. The univariate analysis comparing clinicopathological data in samples with and without *NSD1* mutations was performed using the Fisher’s test. The bivariate logistic regression analysis was performed using the generalized linear model pN = 0.061×TB − 1.014 × NSD1-status − 0.086, where pN represents the lymph node status (metastasis vs no metastasis) as the dependent variable, TB indicates the tumor budding (independent variable), and NSD1-status refers to *NSD1* mutation status (independent variable).

Differential gene expression analysis comparing budding with non-budding tumors was performed using the TCGA-HNSC and CPTAC-HNSCC RNA-Seq raw counts and DESeq2 v.1.42.0^81^. The CPTAC-HNSCC cohort was used to validate the results detected in the analysis of the HPV-negative TCGA-HNSC cohort. A differentially expressed gene was considered validated if it was differentially expressed in the same direction (up- or downregulation) with a one-sided p-value smaller than 0.05. Moreover, the correlation of the level of TB and gene expression was assessed using Spearman correlations in the subset of budding tumors. We then performed functional analyses using the cancer hallmark catalog of MSigDB v7.5^91–93^ and the differentially expressed genes (DEGs) and significantly correlating genes (SCGs). For each gene set, an enrichment fold change of the percentages score was calculated using the formula FC = (*k*/*K*)/(*n*/*N*), in which *k* refers to the number of genes in the MSigDB gene set, *K* refers to the number of DEGs or SCGs, *n* refers to the number of common genes between the *k* and *K* sets, and *N* refers to the number of genes in the respective MSigDB catalog. The enrichment analysis was conducted twice for each analysis; once using the upregulated DEGs/positive SCGs and then using the downregulated DEGs/negative SCGs. The enrichment of the functional categories in the cancer hallmark catalog was assessed using Fisher’s test. Differential miRNA expression (TCGA-HNSC miRNA) and differential protein expression (TCGA-HNSC RPPA, CPTAC-HNSCC MS, TUM-IHC, and TUM-Proteomics) between budding and non-budding tumors were assessed using the Wilcoxon test. H-scores (TUM-IHC cohort) of the bulk and the budding regions of the budding cases were compared using the paired Wilcoxon test. The correlation of the level of TB and protein expression in the subset of budding tumors was analyzed using Spearman correlations. Multiplicity in hypothesis testing was addressed by correcting the p-values using the Benjamini–Hochberg method. Lists of differentially expressed genes (DEGs) and differentially expressed proteins (DEPs) were generated controlling the false discovery rate (FDR) at 10% and including only genes/proteins with absolute fold change above 1.5. Lists of significantly correlating genes (SCGs) were generated controlling the false discovery rate (FDR) at 10% and including only genes with |Spearman *ρ* | ≥ 0.25. We calculated the intersections of these gene lists with the significance assessed using the Fisher’s test and the results were visualized using venn diagrams (R packages GeneOverlap v1.38.0 and VennDiagram v1.7.3). In addition, we calculated the intersection of the above gene lists with the common genes within the p-EMT program as defined in Puram et al.^22^, using the top 15 genes.

Gene and protein expression patterns for selected gene sets were visualized in heatmaps. For the underlying hierarchical clusterings, the similarity of samples or genes was assessed using Pearson correlations and the distance between clusters was calculated using the average linkage method. The following gene sets were analyzed: the EMT and myogenesis MSigDB hallmark gene sets, a combined EMT/myogenesis gene set, and a p-EMT gene set that includes well-established epithelial and mesenchymal markers (epithelial markers: *CDH1*, *CLDN1*, *CLDN2*, *CLDN3*, *CLDN4*, *CLDN5*, *CLDN6*, *CLDN7*, *CLDN8*, *CLDN9*, *CLDN10*, *CLDN11*, *CLDN12*, *CLDN14*, *CLDN15*, *DSP*, *KRT6A*, *KRT8*, *KRT16*, *KRT18*, *KRT19*, and *OCLN*; mesenchymal markers: *ACTA2*, *CDH2*, *FN1*, *GCLC*, *ITGB1*, *ITGB3*, *ITGB6*, *MMP2*, *MMP3*, *MMP9*, *TWIST1*, *VIM*, *VTN*, *ZEB1*, and *ZEB2*; HNSCC-specific p-EMT markers: *CDH1*, *ITGA5*, *SNAI2*, and *VIM*^20,94–97^. For each heatmap, the tumors were grouped in two clusters according to the highest hierarchy in the dendrogram and the difference of TB between the clusters was assessed using Fisher’s test and Wilcoxon test. For the combined EMT/myogenesis gene set, we identified three clusters in the TCGA-HNSC negative subcohort and the differences of TB in the three clusters was assessed using the Kruskal–Wallis test and ad-hoc pairwise Wilcoxon tests. Similarly, for the p-EMT gene set, we identified five clusters in the TCGA-HNSC negative subcohort and the differences of TB in the five clusters was assessed using the Kruskal–Wallis test and ad-hoc pairwise Wilcoxon tests.

Differences in progression-free interval (PFI) and overall survival (OS) were analyzed using Kaplan–Meier curves (R package survminer 0.4.9) and assessed for significance by the log rank-test (R package survival v3.5_7).

For the interactions network analysis, we calculated the Spearman correlations between the expression values of all DEGs or DEPs, as well as the area under the curve (AUC) of the receiver operating characteristic curve (R package pROC v1.18.5) when separating budding and non-budding cases based on the expression of each gene or protein. We then selected those DEGs or DEPs with |Spearman *ρ* | ≥ 0.6 and AUC ≥ 0.7 for visualization. The network graph was generated using Cytoscape (v3.10.2)^98^. Transcription factors (extracted from The Human Transcription Factors database (v1.01, accessed on 15.05.2024)^99^ and EMT and myogenesis MSigDB hallmark genes were annotated in the graph. The genes/proteins in the network were additionally annotated to the MSiGDB gene sets GOBP_IMMUNE_RESPONSE and GOBP_EPIDERMAL_GROWTH_FACTOR_RECEPTOR_SIGNALING_PATHWAY.

The analysis was conducted for the TCGA-HNSC HPV-negative DEGs, the TCGA-HNSC HPV-positive DEGs, and the TUM-LC-MS HPV-negative DEPs.

Many of the analyses included a multitude of molecular features (mutations, miRNAs, mRNAs, and proteins) or functional categories. We strictly addressed the multiplicity in hypothesis testing in each of the analyses by correcting the *P* values using the Benjamini–Hochberg method and controlling the FDR at 10%.

## Supplementary information

Supplementary info

## Data Availability

The TCGA and CPTAC data are available from public repositories, as indicated. The in-house mass spectrometry proteomics data have been deposited to the ProteomeXchange Consortium via the PRIDE^100^ partner repository with the dataset identifier PXD058014. The IHC data are available from the corresponding author on reasonable request.

## Code availability

Statistical analyses and graphics generation were performed using the statistical programming language R v4.3.2. The following packages were used to generate graphics: ggplot2 v3.4.4, ggbeeswarm v0.7.2, ggdist v3.3.1, ggrepel v0.9.4, plotrix v3.8_4, RColorBrewer v1.1_3, amap v0.8_19, and heatmap3 v1.1.9. The code is available from the corresponding author upon request.
