# Supplementary material for: Multi-omics analysis to uncover the molecular basis of tumor budding in head and neck squamous cell carcinoma
Source: NPJ Precis Oncol. 2025 Mar 13;9:73. doi: 10.1038/s41698-025-00856-2 (PMC11906922; doi:10.1038/s41698-025-00856-2)
Supplement: Supplementary file 1 — Supplementary info [file 41698_2025_856_MOESM1_ESM.pdf]

Supplementary Table 1. Analyzed HNSCC cohorts and sample sizes.

Cohort	Data	n_{total}	$n_{tumor\text{-}budding}$	$n_{non\text{-}budding\ cases}$
TCGA-HNSC HPV-	mutations	282	247 (88%)	35 (12%)
TCGA-HNSC HPV+	mutations	30	17 (57%)	13 (43%)
TCGA-HNSC HPV-	miRNA	289	253 (88%)	36 (23%)
TCGA-HNSC HPV+	miRNA	32	20 (63%)	12 (37%)
TCGA-HNSC HPV-	transcriptomics	292	256 (88%)	36 (12%)
TCGA-HNSC HPV+	transcriptomics	33	20 (61%)	13 (39%)
TCGA-HNSC HPV-	RPPA protein expression	143	126 (88%)	17 (12%)
TCGA-HNSC HPV+	RPPA protein expression	11	5 (45%)	6 (55%)
TUM-IHC HPV-	IHC protein expression	21	13 (62%)	8 (38%)
TUM-IHC HPV+	IHC protein expression	20	8 (40%)	12 (60%)
TUM-LC-MS HPV-	MS protein expression	104	104 (67%)	34 (33%)
CPTAC-HNSCC HPV-	transcriptomics	89	66 (74%)	23 (26%)
CPTAC-HNSCC HPV-	MS protein expression	89	66 (74%)	23 (26%)

Supplementary Table 2. **Univariate analysis of the NSD1 mutation status with clinicopathological characteristics**. Significant associations ($p < 0.05$) are highlighted in bold.

**Parameter**		***NSD1* non-mut.**	***NSD1* mut.**	**p-value**
**Age**	≤61	124 (85.5%)	21 (14.5%)	0.7
	>61	120 (87.6%)	17 (12.4%)	
**Sex**	Female	68 (90.7%)	7 (9.3%)	0.2
	Male	176 (85%)	31 (15%)	
**Smoking**	Non-smoker	88 (95.7%)	4 (4.3%)	**0.002**
	Smoker	149 (82.3%)	32 (17.7%)	
	NA	7	2	
**AJCC Stage**	I	12 (92.3%)	1 (7.7%)	1
	II	34 (87.2%)	5 (12.8%)	
	III	34 (82.9%)	7 (17.1%)	
	IV	148 (86.5%)	23 (13.5%)	
	NA	16 (88.9%)	2 (11.1%)	
**Grade**	G1	39 (92.9%)	3 (7.1%)	0.4
	G2	156 (86.7%)	24 (13.3%)	
	G3/G4	47 (81%)	11 (19%)	
	NA	2	0	
**Subtype**	basaloid	7 (77.8%)	2 (22.2%)	0.3
	keratinizing	211 (87.6%)	30 (12.4%)	
	non-keratinizing	26 (81.2%)	6 (18.8%)	
**pN**	N0	81 (77.1%)	24 (22.9%)	**0.002**
	N1/2/3	131 (91.6%)	12 (8.4%)	
	NA	32	2	
**pT**	T1/T2	83 (90.2%)	9 (9.8%)	0.4
	T3/T4	150 (84.3%)	28 (15.7%)	
	NA	11	1	
**cpM**	M0	237 (86.8%)	36 (13.2%)	0.3
	M1	1 (50%)	1 (50%)	
	NA	6	1	
**Localization**	Hypopharynx	5 (100%)	0 (0%)	**0.0001**
	Larynx	56 (70.9%)	23 (29.1%)	
	Oral cavity and lips	170 (92.4%)	14 (7.6%)	
	Oropharynx	13 (92.9%)	1 (7.1%)	
**L1**	absent	240 (86.6%)	37 (13.4%)	0.5
	present	4 (80%)	1 (20%)	
**Pn1**	absent	189 (85.9%)	31 (14.1%)	0.7
	present	55 (88.7%)	7 (11.3%)	
**Margin status**	negative/close	202 (86.3%)	32 (13.7%)	1
	positive	29 (87.9%)	4 (12.1%)	
	NA	13	2	

a

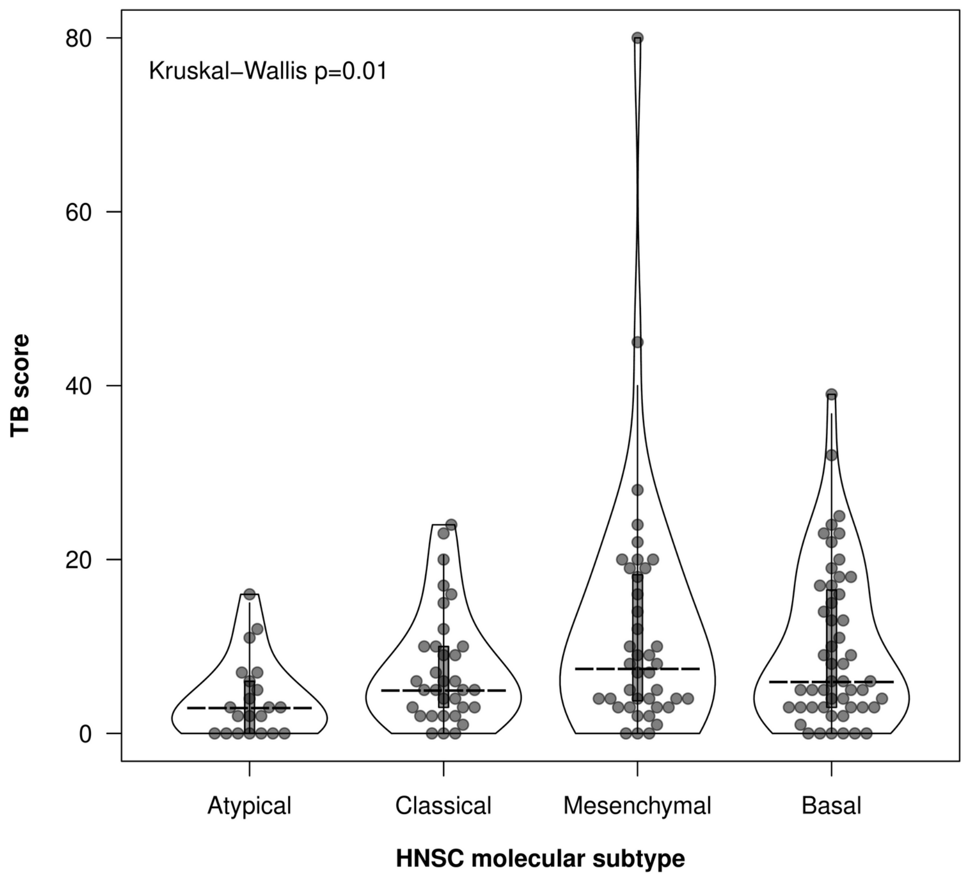

b

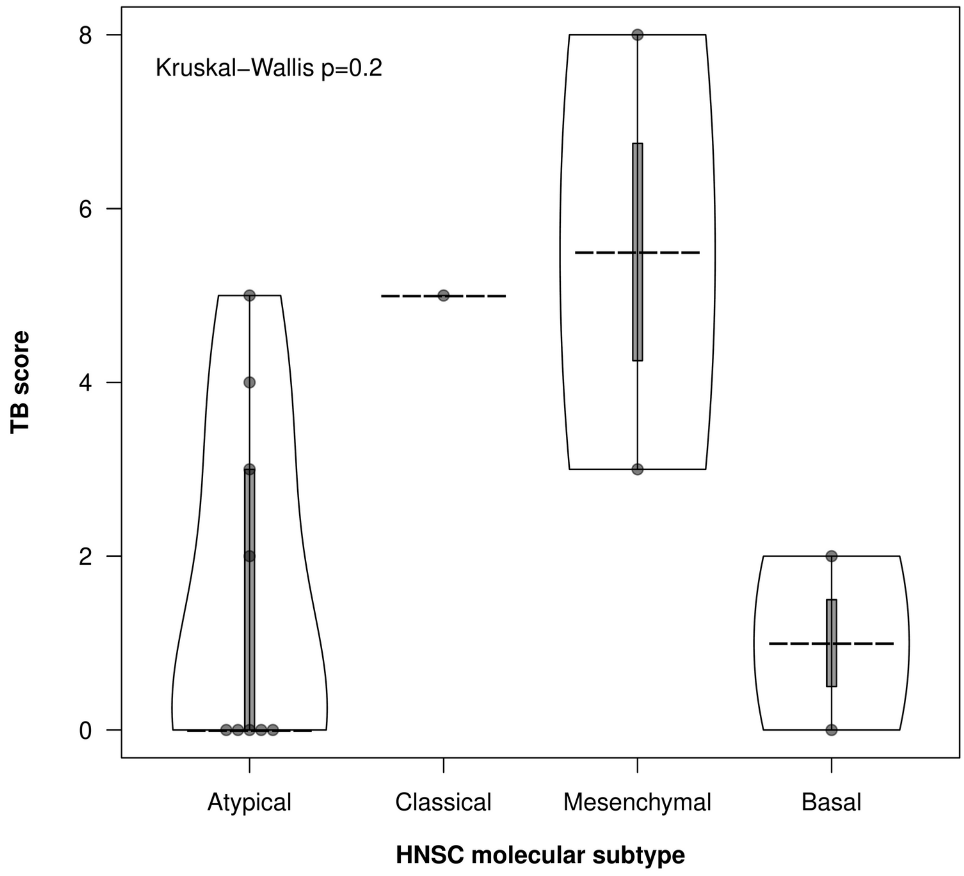

Supplementary Figure 1. Association of TB with the molecular subtypes of HNSCC (TCGA-HNSC data). a HPV-negative HNSCC. **b** HPV-positive HNSCC.

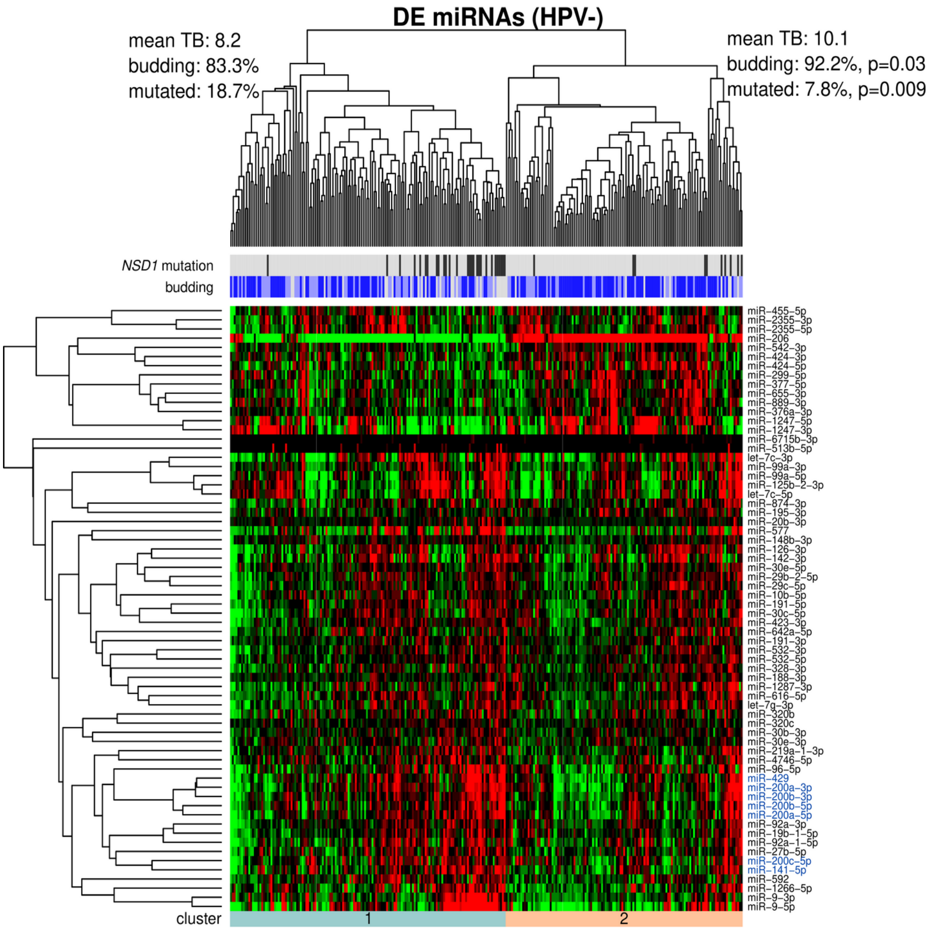

Supplementary Figure 2. Heatmap of differentially expressed miRNAs between budding and non-budding HPV-negative tumors. Significantly different TB and proportion of *NSD1* mutations between the two main tumor clusters. Blue font: miR-200 family.

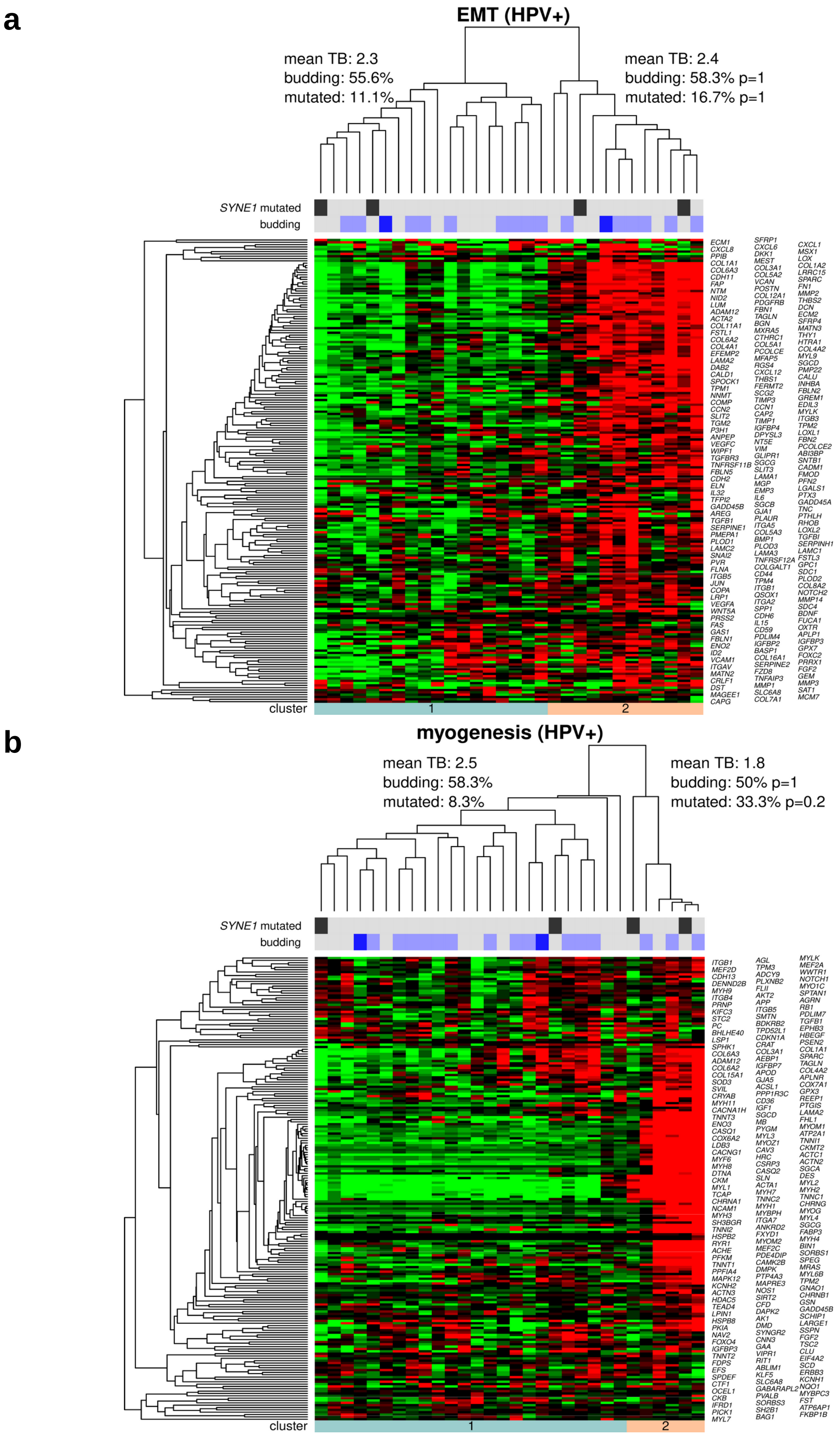

Supplementary Figure 3. Unsupervised gene expression heatmap of the MSiGDB EMT hallmark (**a**) and myogenesis hallmark (**b**) in HPV-positive HNSCC (TCGA cohort). Samples exhibiting tumor budding are marked in light (0 < TB < 6) and dark blue (TB ≥ 6). Samples with mutations in *SYNE1* are shown in dark gray. The samples are grouped in two clusters by dichotomizing the dendrogram at root (orange dashed line). The percentages of the non-budding cases and the cases with mutations are shown on the respective side of the dendrogram, with the p-value representing the result of the fisher's test to test for differences in the percentages in these two clusters.

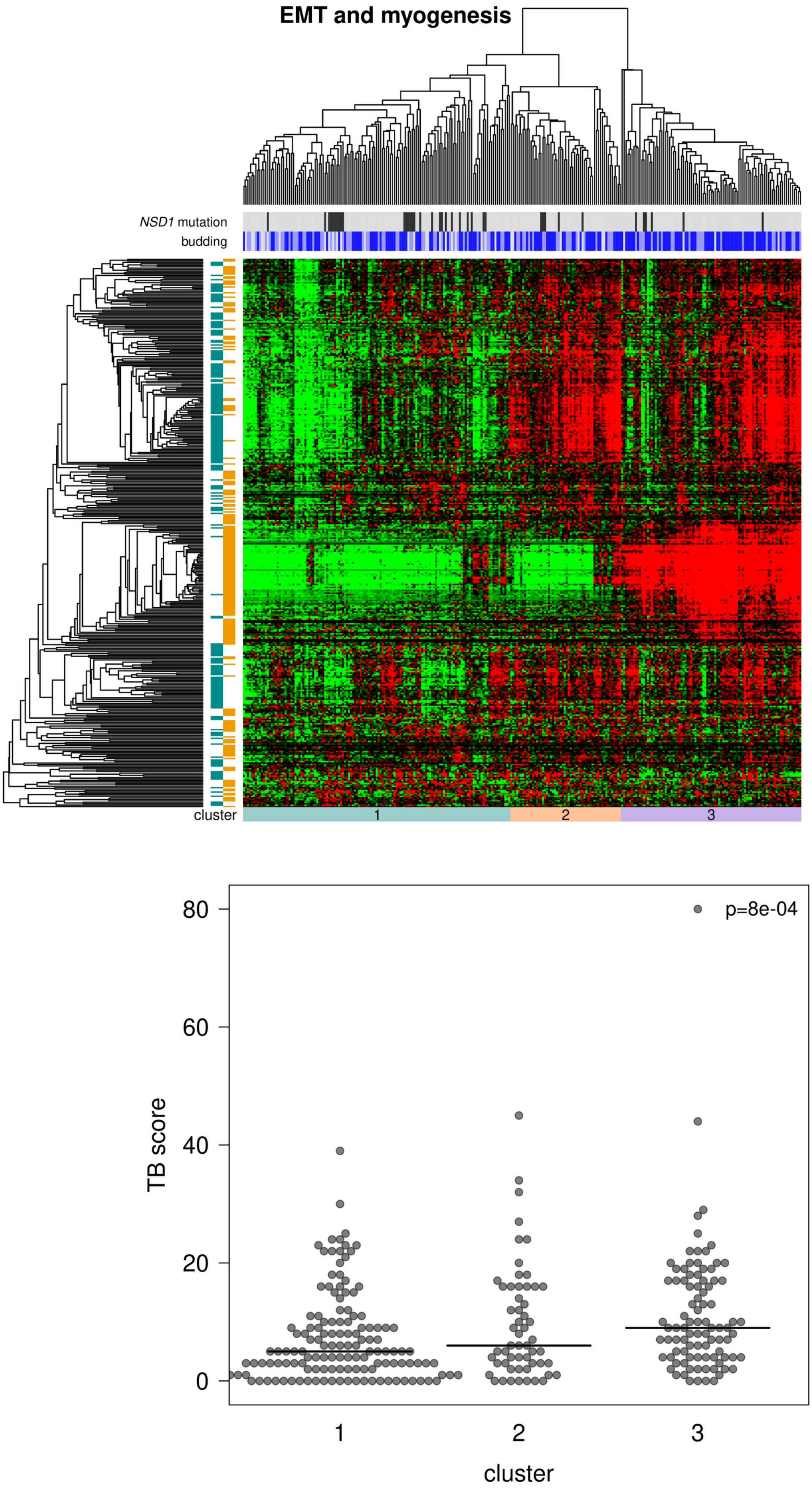

Supplementary Figure 4. Unsupervised clustering of HPV-negative HNSCC with respect to the combined EMT and myogenesis hallmark gene sets. Below the clustering, the TB in the three main clusters is shown in a beeswarm plot.

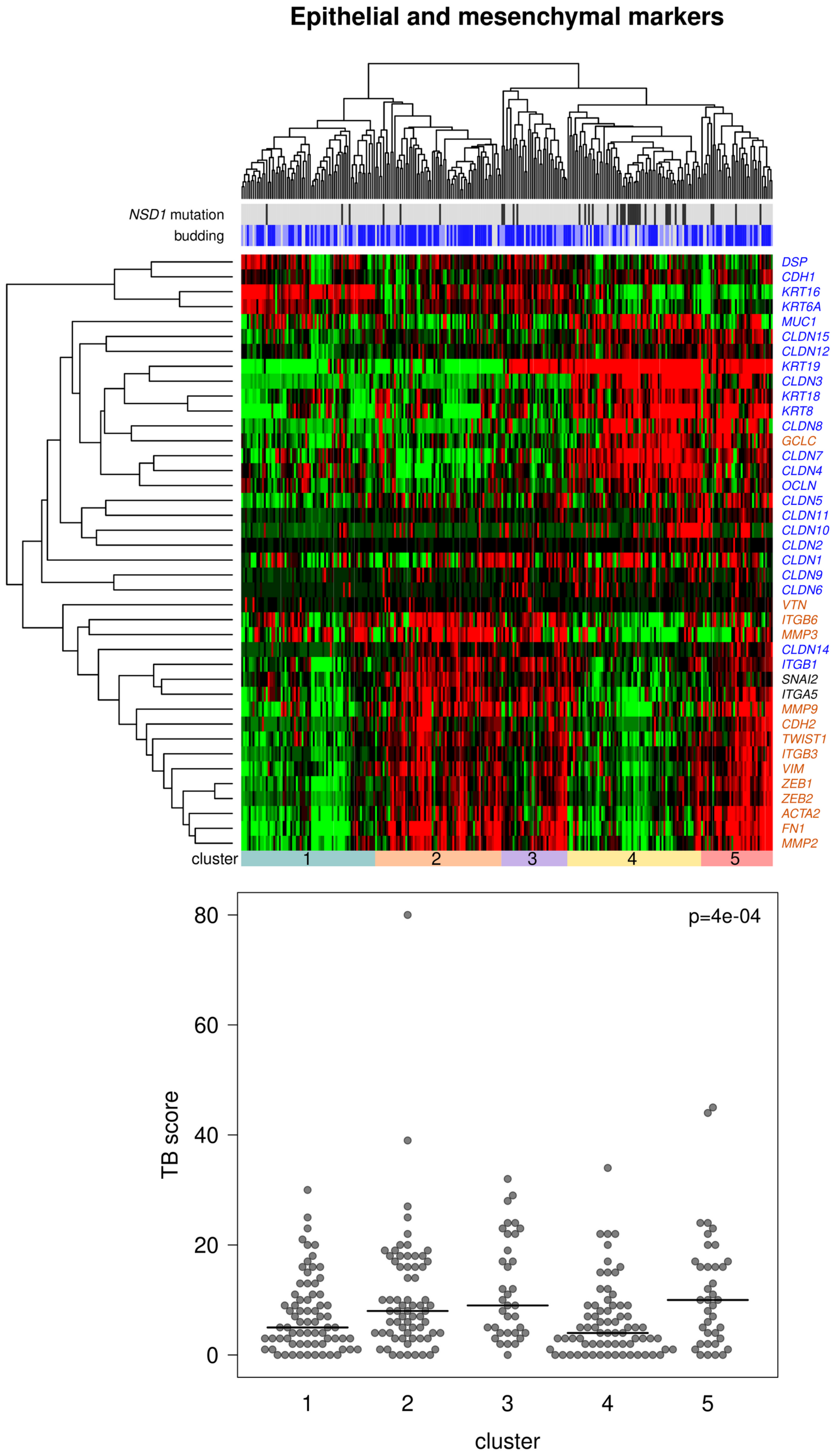

Supplementary Figure 5. Unsupervised clustering of HPV-negative HNSCC with respect to epithelial (blue) and mesenchymal (orange) markers (HPV-negative subcohort). Below the clustering, the TB in the five main clusters is shown in a beeswarm plot. Co-expression of the genes in black (*SNAI2* and *ITGA5*) along with *VIM* and *CDH1* has been reported as a HNSCC-specific pEMT marker (Pal et al. 2021).

Epithelial and mesenchymal markers

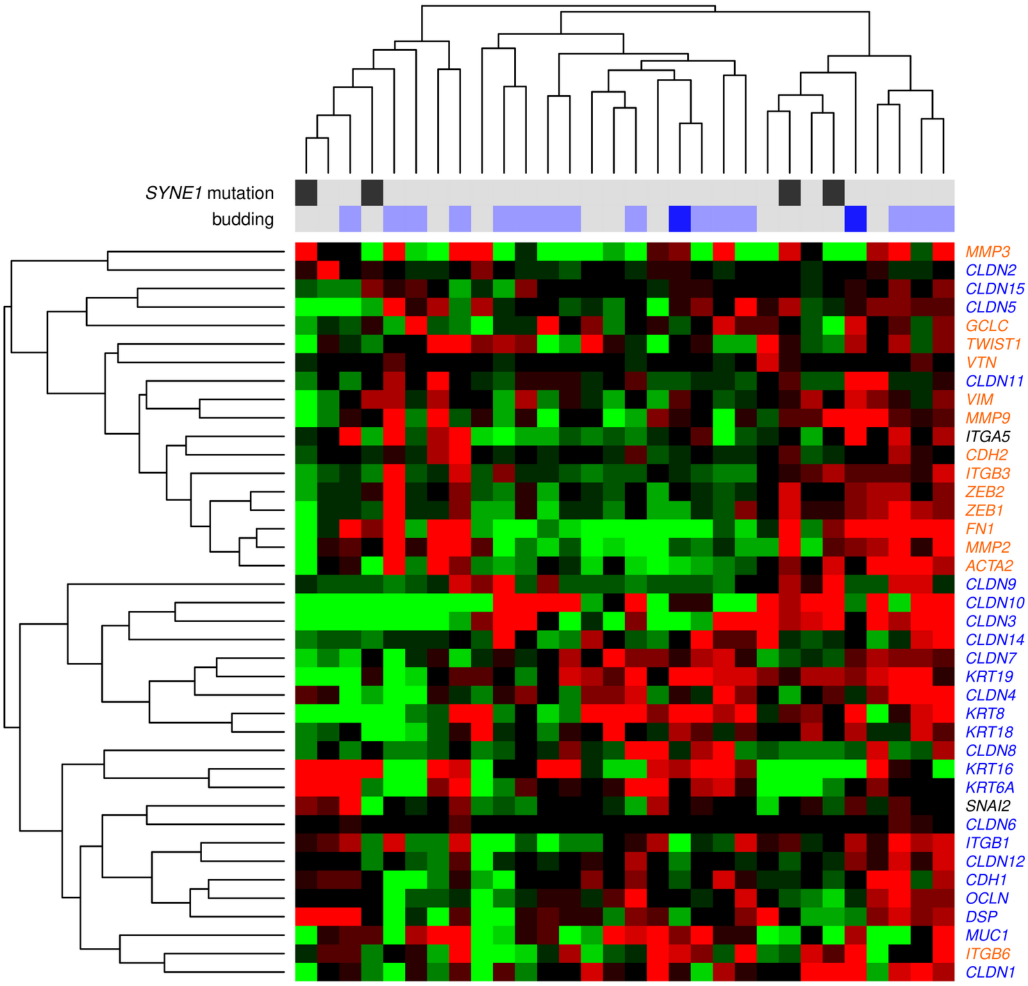

Supplementary Fig. 6. Unsupervised clustering of HPV-positive HNSCC with respect to epithelial (blue) and mesenchymal (orange) markers. Co-expression of the genes in black (*SNAI2* and *ITGA5*) along with *VIM* and *CDH1* has been reported as a HNSCC-specific pEMT marker (Pal et al. 2021).

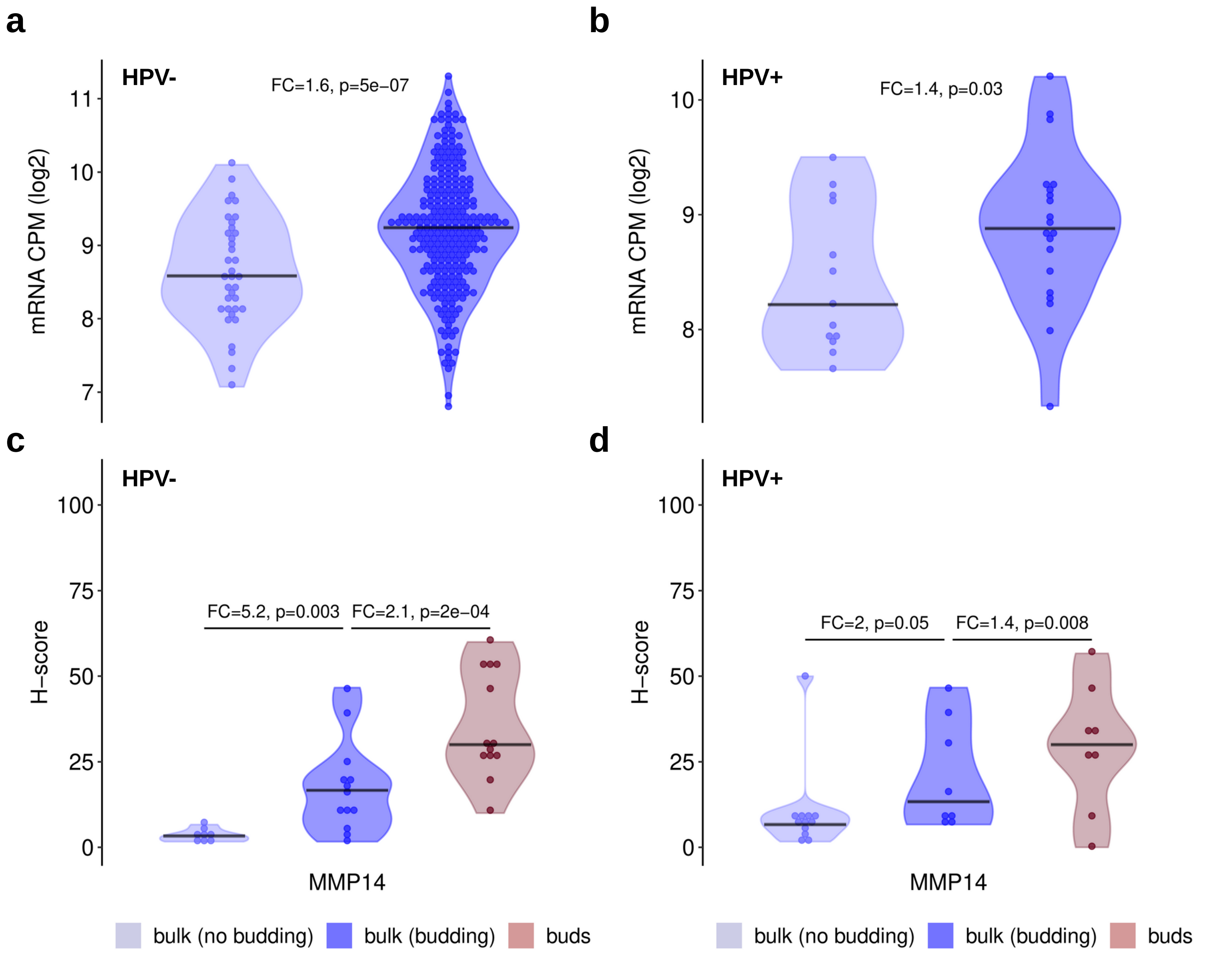

Supplementary Figure 7. Comparison of the expression levels of MMP14 at the gene and protein level in non-budding cases, tumor-budding cases, and in the tumor buds. Gene expression difference between tumor budding and non-budding cases of the TCGA cohort (**a**: HPV-negative, **b**: HPV-positive). Protein expression difference between the bulk of the tumor budding cases and the bulk of the non-budding cases, as well as the bulk of the tumor budding cases and the tumor buds (in-house IHC cohort, **c**: HPV-negative, **d**: HPV-positive). FC: fold change of the means.

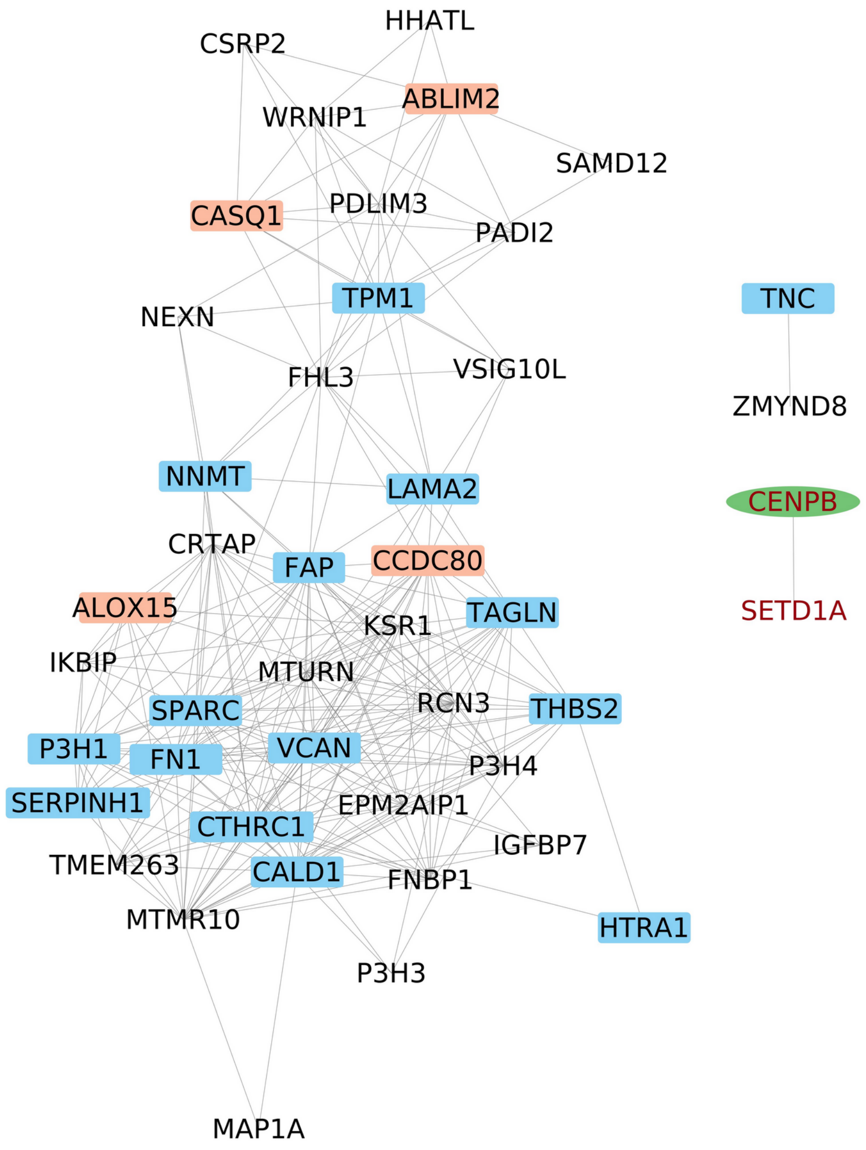

Supplementary Figure 8. Correlation network of the strongly DEP between budding and non-budding tumors (TUM-LC-MS data). Proteins were included in the networks when being significant after multiple testing correction (FDR=10%) and showing both |FC| ≥ 1.5 and AUC ≥ 0.7. Proteins were connected by an edge when correlating with |Spearman *ρ*| ≥ 0.6. Black node font: overexpressed protein, red node font: underexpressed protein. Green ellipse: transcription factor, blue rectangle: EMT protein. Grey edge: positive correlation, red edge: negative correlation. Only proteins correlating above the threshold with at least one other protein are shown.

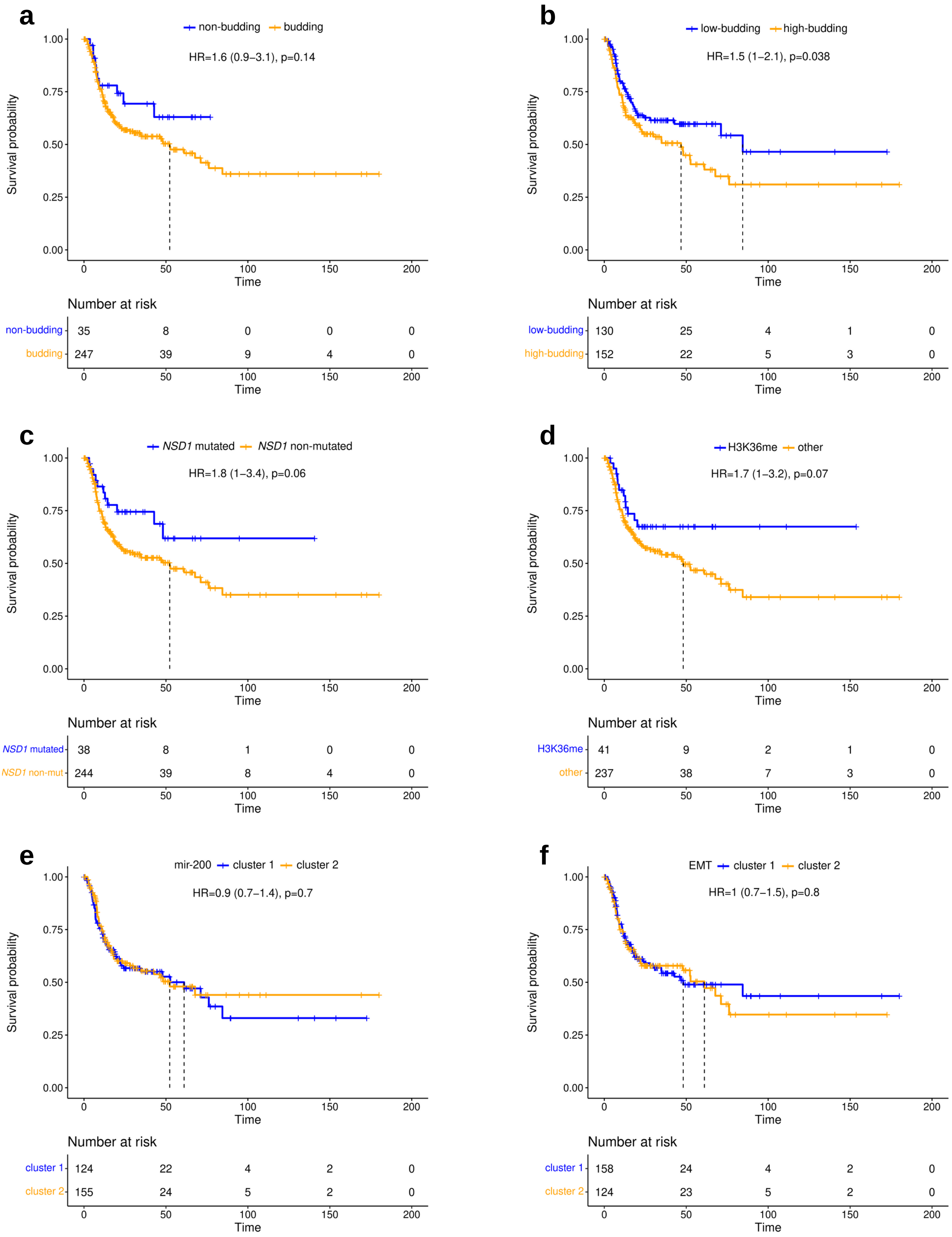

Supplementary Figure 9. Kaplan-Meier curves showing the PFI of the tumor-budding and non-budding cases (**a**), the low budding (TB score <6) and high budding (TB score ≥ 6) cases (**b**), the *NSD1* mutated and non-mutated cases (**c**), the EMT clusters 1 and 2 (**d**), the H3K36me and non-methylated cases (**e**), and the miR-200 clusters 1 and 2 (**f**) in the TCGA-HNSC HPV-negative subcohort. Time is shown in months.

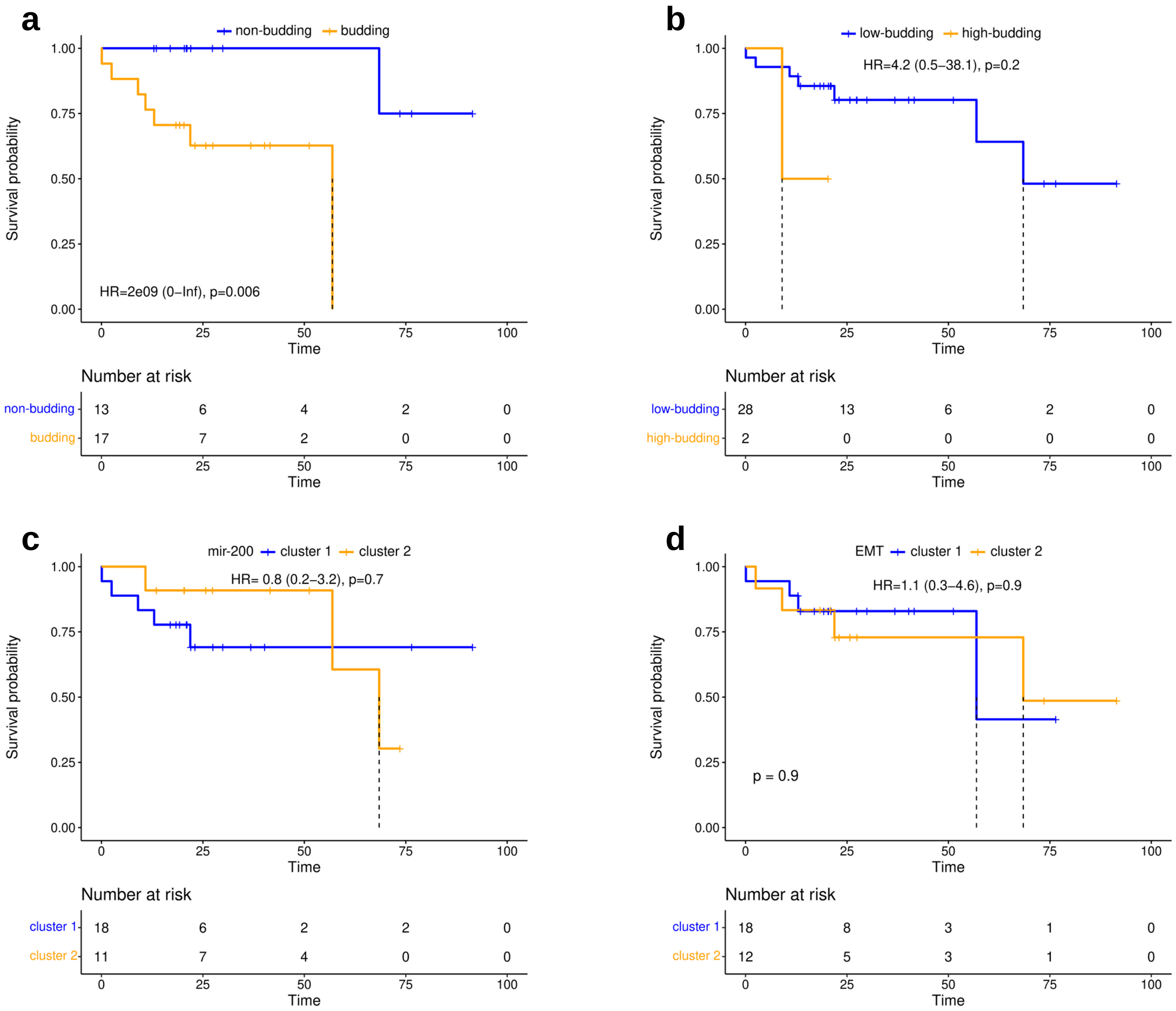

Supplementary Figure 10. Kaplan-Meier curves showing the OS of the tumor budding and non-budding cases (**a**), the low budding (TB score <6) and high budding (TB score ≥ 6) cases (**b**), the miR-200 clusters 1 and 2 (**c**), and the EMT clusters 1 and 2 (**d**) in the TCGA-HNSC HPV-positive subcohort. Time is shown in months.

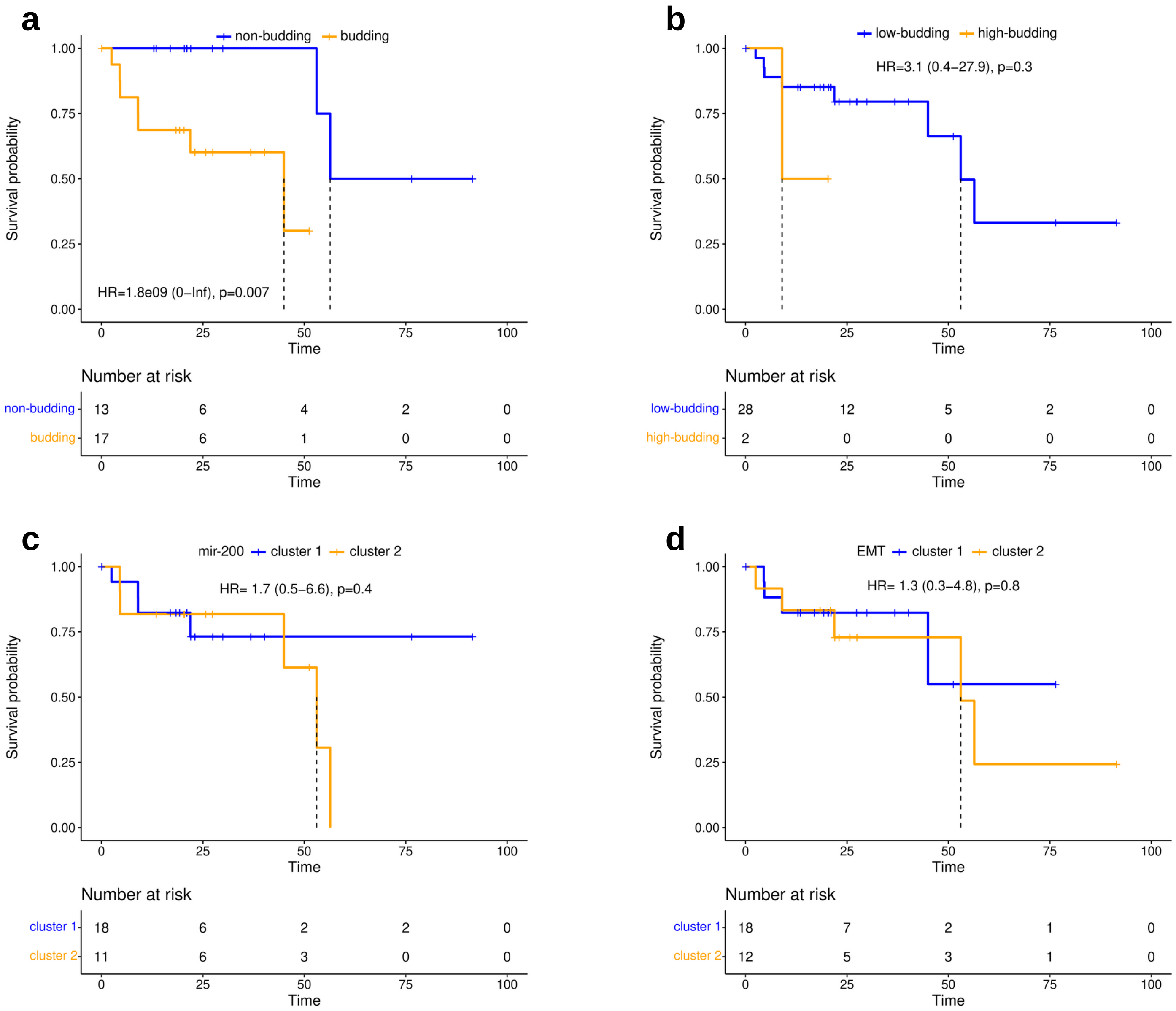

Supplementary Figure 11. Kaplan-Meier curves showing the PFI of the tumor budding and non-budding cases (**a**), the low budding (TB score <6) and high budding (TB score ≥ 6) cases (**b**), the miR-200 clusters 1 and 2 (**c**), and the EMT clusters 1 and 2 (**d**) in the TCGA-HNSC HPV-positive subcohort. Time is shown in months.

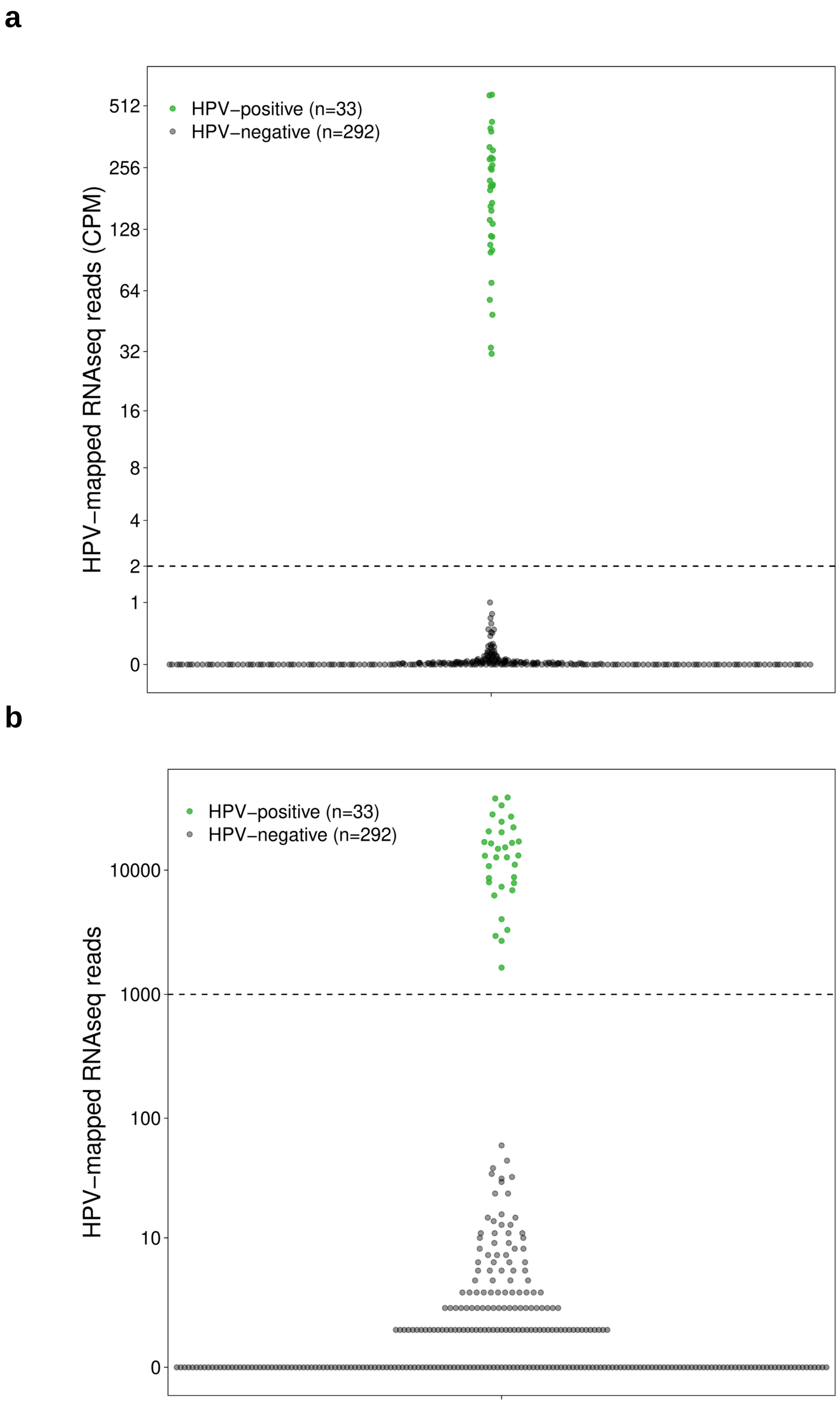

Supplementary Figure 12. Separation of HPV-positive and HPV-negative HNSCC by the number of RNA-seq reads mapped to the HPV genome (TCGA-HNSC cohort). a Separation by CPM-normalized read counts. **b** Separation by raw read counts. Usage of CMP-normalized and raw read counts result in the same classification of the tumors.

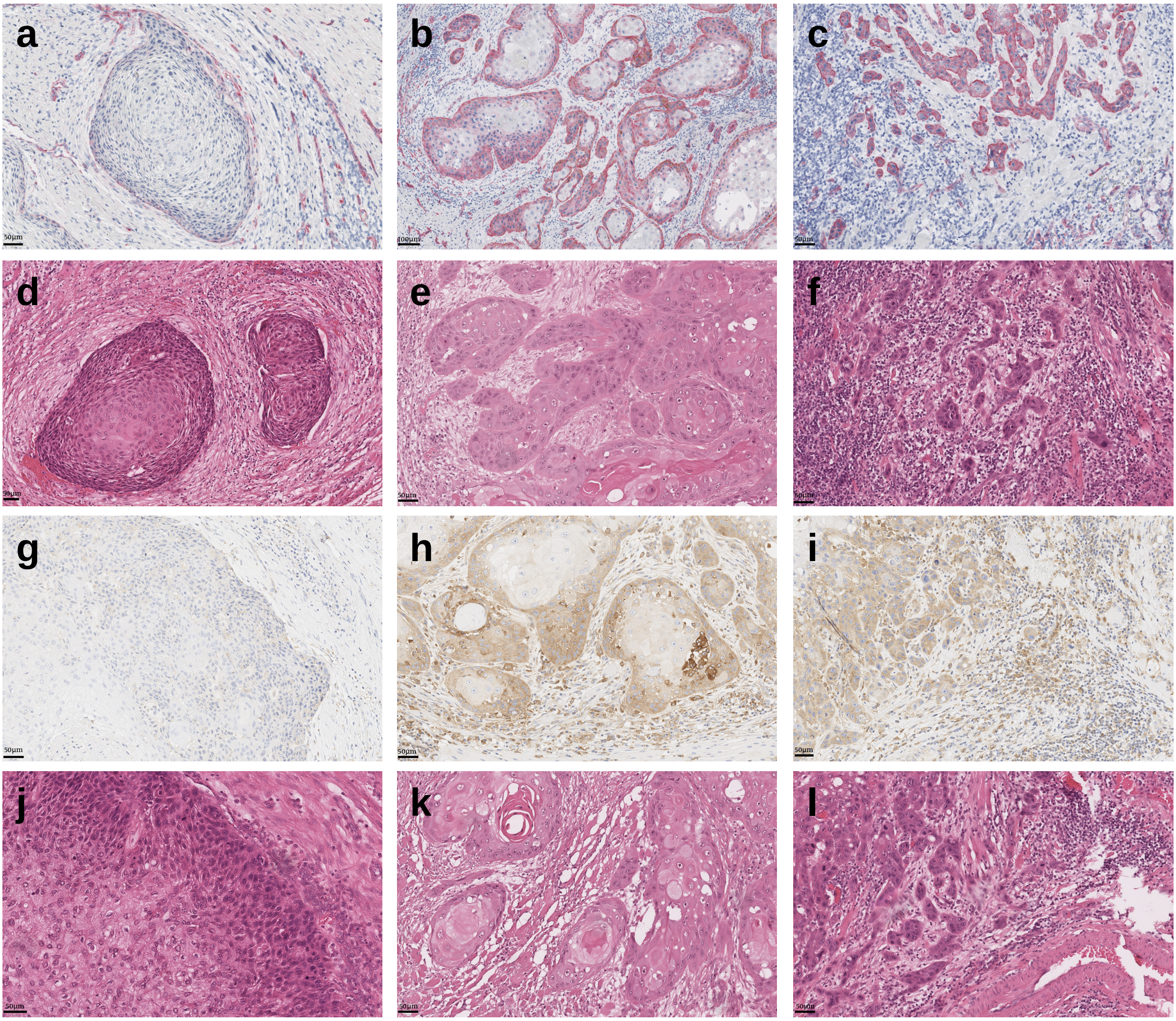

Supplementary Figure 13. Example Caveolin-1 (a, b, c) and Matrix metalloproteinase-14 (g, h, i) IHC stains and the accompanying HE stains (d, e, f, j, k, l) of non-budding samples (a, d, g, j), of the tumor bulk of budding samples (b, e, h, k), and of tumor budding regions of budding samples (c, f, i, l). A “histo-score” (H-score) was calculated as the intensity of staining multiplied by the percentage of cells staining negative (0), weak (1+), moderate (2+), and strong (3+).
